# The effect of omitted covariates in marginal and partially conditional recurrent event analyses

**DOI:** 10.1007/s10985-018-9430-y

**Published:** 2018-05-16

**Authors:** Yujie Zhong, Richard J. Cook

**Affiliations:** 1 MRC Biostatistics Unit, School of Clinical Medicine, University of Cambridge, Cambridge Institute of Public Health, Forvie Site, Robinson Way, Cambridge, CB2 0SR UK; 2 Department of Statistics and Actuarial Science, University of Waterloo, 200 University Avenue West, Waterloo, ON N2L 3G1 Canada

**Keywords:** Asymptotic bias, Confounding, Marginal, Partially conditional, Rate function, Recurrent events

## Abstract

There have been many advances in statistical methodology for the analysis of recurrent event data in recent years. Multiplicative semiparametric rate-based models are widely used in clinical trials, as are more general partially conditional rate-based models involving event-based stratification. The partially conditional model provides protection against extra-Poisson variation as well as event-dependent censoring, but conditioning on outcomes post-randomization can induce confounding and compromise causal inference. The purpose of this article is to examine the consequences of model misspecification in semiparametric marginal and partially conditional rate-based analysis through omission of prognostic variables. We do so using estimating function theory and empirical studies.

**Electronic supplementary material:**

The online version of this article (10.1007/s10985-018-9430-y) contains supplementary material, which is available to authorized users.

## Introduction

Much research has been carried out in the past 20 years on statistical methods for the analysis of recurrent events to better understand chronic disease processes in observational settings and to evaluate the effect of experimental interventions in clinical trials. Disease processes in which recurrent events are manifest are ubiquitous and include, for example, chronic obstructive pulmonary disease where individuals experience recurrent exacerbations (Grossman et al. 1998), epilepsy where seizures recur (Musicco et al. 1997), and cancer where skeletal metastases and associated clinical complications can recur over time (Hortobagyi et al. 1996).

In clinical trials it is essential that tests for treatment effects be valid such that the rejection rate under the null hypothesis is at the nominal level. It is also critically important that models and methods of estimation be formulated so that estimators are consistent for an estimand with a clear causal interpretation. Finally, standard errors must adequately reflect the sampling variation so that confidence intervals have empirical coverage rates that are compatible with the nominal level in finite samples. These criteria form the basis for the following investigation which we carry out in both the clinical trial and observational settings. We confine our attention to marginal rate-based and partially conditional rate-based analyses since these are frequently applied in practice.

Semiparametric models based on marginal rate functions (Andersen and Gill 1982) are among the most widely used for assessing treatment effects on recurrent event processes in clinical trials (Cook and Lawless 2007). Partially conditional models involve time-dependent stratification on the cumulative number of events; this is formulated like a Markov model and is sometimes referred to as the *Prentice–Williams–Peterson* approach, although Prentice et al. (1981) did not advocate its use in clinical trials. It is also often called the *stratified Andersen–Gill* approach due to its relation with the rate-based method of Andersen and Gill (1982). We use the term *partially conditional* model to reflect the fact that, in contrast to intensity-based models, here only part of the process history is conditioned upon. This partially conditional approach has been shown to provide some protection against extra-Poisson variation when model-based variance estimates are used (Boher and Cook 2006), and to mitigate biases induced by event-dependent censoring (Cook et al. 2009). We explore the robustness of the marginal and partially conditional model by evaluating the limiting value and variance of estimators of covariate effects when a Poisson model is misspecified through the omission of a covariate; we consider both the observational and clinical trial setting where interest lies in the effect of a treatment. Performance of these methods when the recurrent events are generated by a multistate Markov process is also considered empirically.

The remainder of the paper is organized as follows. In Sect. 2 we define and give the associated estimating equations for the multiplicative model based on the marginal rate function (Andersen and Gill 1982) as well as the partially conditional model (Prentice et al. 1981). The limiting behaviour of estimators of treatment effect are given in Sect. 3 for the marginal and partially conditional models when the events are generated by a Poisson process but a prognostic covariate is omitted. The results of empirical studies supporting the large sample theory are given in Sect. 4 where the investigation is broadened to study the setting where events are generated by a Markov process but a covariate is omitted in the marginal and partially conditional analyses. An application illustrating the various methods is given in Sect. 5 and concluding remarks are given in Sect. 6.

## Marginal and partially conditional rate-based models 

### Multiplicative models based on marginal rate functions

Let $N_i(t)$ denote the number of events occurring over [0, *t*] and $\{N_i (t), 0 \le t\}$ be the right-continuous counting process for individual *i* in a sample of *n* independent individuals, $i=1, 2, \ldots , n$. The number of events over the interval $[t, t+\Delta t)$ for individual *i* is then $\Delta N_i(t)=N_i(t+\Delta t^-)-N_i(t^-)$ and $dN_i (t) = \lim _{\Delta t \downarrow 0} \Delta N_i (t)$. We let $X_i (t) = (X_{i1}(t), \ldots , X_{ip}(t))'$ denote a $p\times 1$ vector of external potentially time-dependent covariates where the process $\{X_i (t), 0 \le t\}$ is left-continuous. The process history is denoted by ${{{\mathcal {H}}}}_i (t) = \{N_i (s), X_i(s): \, 0 \le s < t \}$.

The stochastic nature of any point process can be characterized by an intensity function,

1 $$\begin{aligned} \lim _{\Delta t \downarrow 0} \frac{P(\Delta N_i (t) = 1| {{\mathcal {H}}}_i (t))}{\Delta t} = \lambda _i (t|{{\mathcal {H}}}_i (t) ) , \end{aligned}$$

which represents the instantaneous probability of an event at time *t* given the process history (Ross 1983; Taylor and Karlin 1984). Of course for a particular setting one must make model assumptions; the canonical model for recurrent events with time-dependent covariates is the modulated Poisson model (Lawless 1987; Cook and Lawless 2007, Chapter 3). The conditionally independent increment property of the modulated Poisson model implies that given $X_i(t)$ the risk at time *t* does not depend on $\{N_i(s), 0 \le s < t\}$, yielding an intensity of the form $\lambda _i (t|{{\mathcal {H}}}_i (t)) = \rho _i (t|X_i (t))$. Multiplicative models with

2 $$\begin{aligned} \rho _i (t|X_i(t);\theta ) = \rho _0 (t; \alpha ) g(X_i (t);\beta ) , \end{aligned}$$

are most common, where $\rho _0 (t; \alpha )$ is a baseline rate function indexed by $\alpha$, $g(X_i(t);\beta )$ is a positive valued function indexed by $\beta$, and $\theta = (\alpha ' , \beta ')'$. Lawless (1987) gives the partial likelihood and associated estimating equations for the semiparametric setting where $\rho _0 (t, \alpha )$ is an arbitrary positive-valued function, and Andersen and Gill (1982) derive the large sample theory; the semiparametric model (2) is sometimes called the Andersen–Gill model. Lin et al. (2000) provide a rigorous derivation of the limiting behaviour of estimators with an emphasis on robust variance estimation.

Individuals are typically followed over a finite period of time to record the occurrence of events of interest. Let the start of the interval be denoted by 0 and *A* denote the planned administrative censoring time. To accommodate early withdrawal we let $R_i$ be a non-negative random variable independent of the recurrent event and covariate process with survivor function $P(R_i \ge t)={{\mathcal {G}}} (t)$, and let $C_i = \min (R_i, A)$ be the effective right-censoring time for individual *i*; the function $Y_i (t) = \text{ I }(t \le C_i)$ indicates whether individual *i* is under observation at time $t>0$, $i=1,\ldots , n$. Under independent and non-informative censoring (Cook and Lawless 2007), the log partial likelihood contribution for individual *i* having $n_i$ events at times $t_{i1}< \cdots < t_{i n_i}$ over $[0, C_i]$ is

3 $$\begin{aligned} \int _0^{\infty } Y_i(t) \left\{ \log \rho _i (t|X_i(t); \theta ) dN_i (t) - \rho _i (t|X_i(t); \theta ) dt \right\} , ~~~ i=1,\ldots , n. \end{aligned}$$

In the semiparametric setting of (2) the function $g(x;\beta ) = \exp (x'\beta )$ is used most often. We let $d\mu _0(t)=\rho _0(t)dt$ ($t>0$) so that $d \mu _0(\cdot )$ can be viewed as an infinite dimensional parameter; differentiating the terms in (3) with respect to $d\mu _0(t)$ we obtain the estimating equations

4 $$\begin{aligned} \sum _{i=1}^{n} Y_i (t)\left\{ dN_i (t) - d\mu _0 (t) \exp (X'_i (t) \beta )\right\} = 0 ,~~~ 0< t . \end{aligned}$$

The profile Breslow-type estimator $d{\tilde{\mu }}_0 (t; \beta ) = d{\bar{N}}_{\cdot }(t)/\sum _{i=1}^n Y_{i}(t) \exp (X'_i (t) \beta )$. is the solution where $d {\bar{N}}_{\cdot }(t) = \sum _{i=1}^{n} Y_i (t) dN_i (t)$. Differentiating (3) with respect to $\beta$ and replacing $d \mu _0(t)$ with $d{\tilde{\mu }}_0 (t; \beta )$ gives the profile partial score equation

5 $$\begin{aligned} U (\beta ) = \sum _{i=1}^n U_i(\beta ) = \sum _{i=1}^n \int _0^{\infty } Y_i (t) \left\{ X_i (t) - \frac{S^{(1)} (\beta , t)}{S^{(0)} (\beta , t)} \right\} dN_i (t) = 0 , \end{aligned}$$

where $S^{(r)} (\beta , t) = n^{-1} \sum _{i=1}^{n} Y_i (t) \exp (X'_i(t) \beta ) X_i(t)^{\otimes r}$ with $X_i(t)^{\otimes 0} = 1$, $X_i(t)^{\otimes 1}=X_i(t)$ and $X_i(t)^{\otimes 2}=X_i(t) X'_i(t)$. Lin et al. (2000) showed that

6 $$\begin{aligned} \sqrt{n} ({\hat{\beta }} - \beta ^\dagger ) \rightarrow \text{ N }\left( 0, {{\mathcal {A}}}^{-1}(\beta ^\dagger ) \mathcal{B}(\beta ^\dagger ) [{{\mathcal {A}}}^{-1}(\beta ^\dagger )]' \right) , \end{aligned}$$

where ${\hat{\beta }}$ is the solution to (5), $\mathcal{A}(\beta ) = E[-\partial U_i (\beta ) / \partial \beta ]$, $\mathcal{B}(\beta ) = E[U_i (\beta )U'_i (\beta )]$, and $\beta ^\dagger$ is the solution to

7 $$\begin{aligned} \int _0^{\infty } E\left[ Y_i (t) \left\{ X_i(t) - \frac{s^{(1)} (\beta , t)}{s^{(0)} (\beta , t)} \right\} dN_i (t)\right] = 0 \end{aligned}$$

where $s^{(r)}(\beta , t) = E[S^{(r)}(\beta , t)]$, $r=0, 1$, and $E[\; \cdot \;]$ denotes an expectation taken with respect to the censoring, recurrent event and covariate processes.

### Multiplicative models based on partially conditional rate functions

A common partially conditional model is obtained by specifying

8 $$\begin{aligned} \lim _{\Delta t \downarrow 0} \frac{P(\Delta N_{ij}(t) = 1 |N_i (t^- ) = j-1, {{\mathcal {H}}}_i (t) )}{\Delta t} = \rho _{j0}(t)g(X_i (t);\beta ) \end{aligned}$$

where $\rho _{j0}(t)$ is an event-specific baseline rate function. The specification in (8) corresponds to a multiplicative intensity-based model for a Markov process with a common treatment effect (Prentice et al. 1981), but otherwise should be viewed as a partially conditional model because only part of the history, namely $N_i(t^-)=j-1$, is conditioned upon.

For convenience in what follows we let $\rho _{j0}(t)g(X_i (t);\beta ) = \rho _{ij}(t|X_i(t))$ and write simply $\rho _{ij}(t)$ to suppress its dependence on $X_i(t)$; $\mu _{ij}(t) = \int _0^t \rho _{ij}(s) ds$. Because $\{X_i(s), 0\le s \}$ is external we can conceive of conditioning on the complete covariate path $\{ X_i(s), 0 \le s\}$ but we will ultimately focus primarily on the case of fixed covariates. We let $Y_{ij}(t) =I(N_i (t^-) = j-1)$ indicate that individual *i* is at risk for their *j*th event at *t* and define ${\bar{Y}}_{ij}(t) = Y_i (t) Y_{ij}(t)$, $i=1, \ldots , n$. We let $dN_{ij}(t) = 1$ indicate the *j*th event for individual *i* occurs at time *t*, and $dN_{ij}(t) = 0$ otherwise; $d{\bar{N}}_{ij}(t) = {\bar{Y}}_{ij}(t) dN_{ij}(t)$ indicates that the *j*th event occurs at *t* and *is observed*.

If individual *i* is observed to experience $n_i$ events at time $t_{i1}< \cdots < t_{i n_i}$ over $[0,C_i]$, the estimating equation for $\beta$ based on a sample of *n* independent individuals is

9 $$\begin{aligned} {\tilde{U}}(\beta ) = \sum _{i=1}^{n} {\tilde{U}}_i(\beta ) = \sum _{i=1}^{n} \sum _{j=1}^{n_i} \int _0^{\infty } {\bar{Y}}_{ij}(t) \left\{ X_i (t) - \frac{S_j^{(1)} (\beta , t)}{S_j^{(0)} (\beta , t)} \right\} dN_{ij}(t), \end{aligned}$$

where here $S^{(r)}_j (\beta , t) = n^{-1} \sum _{i=1}^{n} {\bar{Y}}_{ij}(t) \exp (X'_i(t) \beta ) X_i(t)^{\otimes r}$; this is the profile pseudo-score function for the partially conditional model. Solving ${\tilde{U}}(\beta ) = 0$ yields the estimate ${\tilde{\beta }}$ which has the asymptotic distribution

10 $$\begin{aligned} \sqrt{n} ({\tilde{\beta }} - \beta ^\ddagger ) \rightarrow N(0, \tilde{{{\mathcal {A}}}}^{-1}(\beta ^\ddagger ) \tilde{\mathcal{B}}(\beta ^\ddagger ) [\tilde{{{\mathcal {A}}}}^{-1}(\beta ^\ddagger )]' ), \end{aligned}$$

where $\tilde{{\mathcal {A}}}(\beta ) = E[-\partial {\tilde{U}}_i (\beta ) /\partial \beta ]$, $\tilde{{\mathcal {B}}}(\beta ) = E[{\tilde{U}}_i (\beta ){\tilde{U}}'_i (\beta )]$ and $\beta ^\ddagger$ is the solution to

11 $$\begin{aligned} \sum _{j=1}^{\infty } \int _0^{\infty } E\left[ {\bar{Y}}_{ij}(t) \left( X_i (t) - \frac{s^{(1)}_j (\beta , t)}{s^{(0)}_j (\beta , t)} \right) dN_{ij} (t) \right] = 0 \end{aligned}$$

with $s_j^{(r)} (\beta , t) = E[S_{j}^{(r)}(\beta , t)]$, $r=0, 1$. These expressions to calculate the bias and asymptotic robust variance are very general, and in principle could be used to evaluate the large sample behaviour of estimators for any underlying recurrent event process. Our interest however, is on the effect of omitted covariates and we explore this in detail in the next section.

## Inference regarding treatment effects with omitted covariates

### Asymptotic properties for estimators of treatment effect

Given the general theory reviewed in Sect. 2 we can now explore the limiting behaviour of treatment effect estimators under misspecified marginal and partially conditional models. Here we consider modulated Poisson processes with a binary treatment covariate *X* and an external potentially time-varying covariate *Z*(*t*). The true rate function is assumed to have the form

12 $$\begin{aligned} \rho (t|X, Z(t)) = \rho _0 (t) \, \exp (\eta X + \zeta Z(t) ), \end{aligned}$$

where $\rho _0 (t)$ is a positive-valued baseline rate function. In the setting of a randomized trial $X \perp Z(t)$ since *Z*(*t*) is external, but *X* and *Z*(*t*) may be correlated in the observational setting. When we model just the treatment indicator (i.e. we omit *Z*(*t*)), we fit the marginal rate-based model $\rho (t|X) = \rho _0^{*}(t) \exp (\beta X)$ or the partially conditional model $\rho _{j}(t|X) = \rho _{j0}^{*}(t)\exp (\beta X)$.

From (7) the asymptotic bias of the Andersen–Gill estimator ${\hat{\beta }}$ is $(\beta ^\dagger - \eta )$. To derive the explicit form we note that in the present setting we can evaluate (7) by first taking the expectation with respect to $dN_{i}(t)|Y_i(t), X_i, Z_i(t)$ to obtain $E_{dN_i(t)} \{U_i(\beta )|Y_i(t), X_i, Z_i(t)\}$ as

$$\begin{aligned} \int _0^{\infty } Y_i (t) \left\{ X_i - \frac{s^{(1)} (\beta , t)}{s^{(0)} (\beta , t)} \right\} \exp (\eta X_i + \zeta Z_i (t) ) d\mu _0 (t) \end{aligned}$$

under the assumption of conditionally independent censoring (i.e. $R_i \perp {{\mathcal {H}}}_i(t)$). Then taking the expectation with respect to the remaining terms gives

13 $$\begin{aligned} \int _0^{A} {{\mathcal {G}}}(t) \left\{ E[X_i \exp (\eta X_i + \zeta Z_i (t))] - E[\exp (\eta X_i + \zeta Z_i (t))]\cdot \frac{s^{(1)} (\beta , t)}{s^{(0)} (\beta , t)}\right\} d\mu _0 (t), \end{aligned}$$

where $s^{(r)}(\beta , t) = E[S^{(r)}(\beta , t)] = {{\mathcal {G}}}(t) E[e^{\beta X_i} X^{\otimes r}_i ]$. When $X_i$ is a binary treatment indicator with, say, $P(X_i=1)=0.5$, then $s^{(1)}(\beta , t)/s^{(0)}(\beta , t) = \exp (\beta )/(1 + \exp (\beta ))$ and substituting this into (13) and solving gives

14 $$\begin{aligned} \exp (\beta ^\dagger ) = \frac{\int _0^{A} {{\mathcal {G}}}(t) E[X_i \exp (\eta X_i + \zeta Z_i (t))]d\mu _0 (t)}{\int _0^{A} {{\mathcal {G}}}(t) E\left[ (1-X_i) \exp (\eta X_i + \zeta Z_i (t))\right] d\mu _0 (t)}. \end{aligned}$$

When $Z_i (t)$ is independent of $X_i$ as in a randomized controlled trial, $\beta ^\dagger = \eta$ so a consistent estimate of the causal effect of treatment ($\eta$) is obtained even when an important (external) covariate is omitted. When *Z*(*t*) and *X* are correlated however, a marginal model omitting *Z*(*t*) will yield a biased estimate of the treatment effect with no easy causal interpretation. Finally note that in the case of a fixed covariate $Z_i(t) = Z_i$ which is possibly correlated with $X_i$, (14) can be simplified to

15 $$\begin{aligned} \exp (\beta ^\dagger ) = \frac{E[X_i \exp (\eta X_i + \zeta Z_i)]}{E[(1-X_i)\exp (\eta X_i + \zeta Z_i)]}. \end{aligned}$$

For the partially conditional model (11) gives

$$\begin{aligned} \sum _{j=1}^{\infty }\int _0^{\infty } \left\{ E\left[ {\bar{Y}}_{ij}(t) X_i d\mu _i(t)\right] - E\left[ {\bar{Y}}_{ij}(t)d\mu _i(t)\right] \cdot \left( \frac{s_j^{(1)}(\beta , t)}{s_j^{(0)} (\beta , t)}\right) \right\} \end{aligned}$$

which can be written as

16 $$\begin{aligned} \sum _{j=1}^{\infty }\int _0^{\infty } \left\{ s_j^{(1)}(t) - s_j^{(0)}(t)\cdot \left( \frac{s_j^{(1)}(\beta , t)}{s_j^{(0)} (\beta , t)}\right) \right\} dt, \end{aligned}$$

where $s_j^{(r)} (t) = E[{\bar{Y}}_{ij}(t) \rho _i (t) X_i^{\otimes r}]$; $r=0, 1$. Note that

$$\begin{aligned} s_{j}^{(r)}(t)= & {} E\left[ {{\mathcal {G}}}(t) \cdot P(N_i (t^- ) = j-1 |X_i, Z_i (t) ) \rho _0(t) \exp (\eta X_i + \zeta Z_i (t)) X_i^{\otimes r} \right] \\= & {} {{\mathcal {G}}}(t) \rho _0(t) \cdot E_{X_i, Z_i (t) } \left[ \frac{\exp (-\mu _i (t)) (\mu _i (t))^{j-1}}{(j-1)!} \exp (\eta X_i + \zeta Z_i (t)) X_i^{\otimes r} \right] , \end{aligned}$$

which depends on the event number *j* and time *t* even if $$Z_i(t)$$ is a time-invariant covariate; the same is true for $$s_{j}^{(r)}(\beta , t)$$. As $$Z_i (t)$$ is an external covariate we can condition on it and think of $$\mu _i (t) = \int _0^{t} \rho _i (s) ds$$ as a mean of $$N_i (t)$$ given $$(X_i, \{Z_i (s), 0 \le s < t\})$$. Since there is no solution in closed-form, one must solve equation $$E[{\tilde{U}}(\beta )] = 0$$ numerically for $$\beta ^\ddagger $$. The complexity of the asymptotic calculation arises because of the extra conditioning on $$Y_{ij}(t)$$ in the partially conditional model. In general, $$\beta ^\ddagger \ne \eta $$, even when $$X_i$$ and $$Z_i (t) $$ are independent. This indicates that omitting the covariate *Z*(*t*) in the partially conditional model leads to a biased estimate of the causal treatment effect, even when *Z*(*t*) and *X* are independent. For the partially conditional model one conditions on the cumulative event count at *t* which is responsive to both treatment and other covariate effects, and hence

$$\begin{aligned} X_i \not \perp Z_i(t) |N_i(t^-) = j-1, ~~ t > 0 . \end{aligned}$$

The phenomenon of induced confounding through this conditioning is well-known in causal inference (Hernán 2010).

The model-based naive variance ${{\mathcal {A}}}^{-1}(\beta ^\dagger )$ will underestimate the variability of ${\hat{\beta }}$ under a misspecified marginal model so robust variance estimation is recommended to ensure valid inference (Lin and Wei 1989; Bernardo and Harrington 2001; Boher and Cook 2006). The explicit forms of the model-based naive and robust sandwich variances in the current setting are given in Appendix 1 and 2 for the marginal and partially conditional models respectively.

### A case-study involving an omitted fixed covariate 

Here we consider a case study of the effect of omitting covariates in the marginal and partially conditional models by considering a particular setting in detail. We assume a Poisson process with a Weibull rate function $\rho (t) = \rho _0 (t) \exp (\eta X + \zeta Z)$ with $\rho _0 (t) = \lambda \kappa (\lambda t)^{\kappa - 1}$. We let *X* be a binary treatment indicator with $P(X=1) = P(X=0) = 0.5$ as before, and let *Z* be a fixed binary covariate with $Z \sim \text{ Bin }(1, p_z )$; we let

$$\begin{aligned} \phi = \frac{P(Z=1|X=1)/P(Z=0|X=1)}{P(Z=1|X=0)/P(Z=0|X=0)} \end{aligned}$$

denote the odds ratio characterizing the association between *X* and *Z* where $X \perp Z$ when $\phi = 1$.

**Fig. 1:**
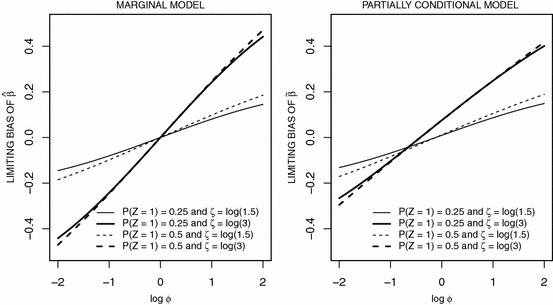
Limiting bias of estimates of treatment effect under the marginal model (left panel) and the partially conditional model (right panel) when omitting the covariate *Z*; *X* and *Z* are dependent binary covariates with odds ratio $\phi$

We let $\beta = \log 0.75$, which reflects the positive effect of treatment and $\zeta = 0$, $\log 1.5$ or $\log 3$ to represent the case of no, moderate or strong effects of *Z* on event occurrence. We let $\kappa = 1.25$, and choose $\lambda$ such that the expected number of observed events at $t=1$ is 2 when *X* and *Z* are equal to 0. Without loss of generality we let the administrative censoring time be $A = 1$ and we assume the random censoring time $R_i$ follows an exponential distribution satisfying $P(R_i < A) = 0.2$; this gives the effective censoring time $C_i = \text{ min }(R_i, A)$. Under this setting, when we omit variable *Z* in the Andersen–Gill model, then by (15) the limiting bias of ${\hat{\beta }}$ is

17 $$\begin{aligned} \beta ^{\dagger } - \eta = \log \left[ \frac{e^{\zeta } P(X=1,Z=1) + P(X=1,Z=0)}{e^{\zeta } P(X=0,Z=1) + P(X=0,Z=0) }\right] \; \end{aligned}$$

**Fig. 2:**
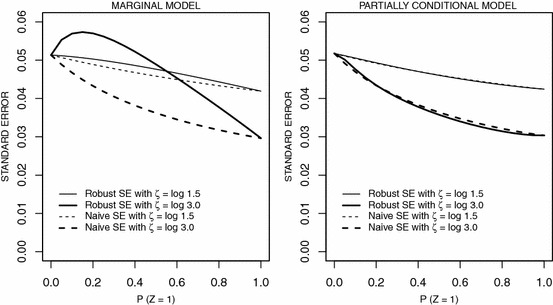
Asymptotic naive and robust standard errors of estimates of treatment effect under the marginal model (left panel) and partially conditional model (right panel) omitting a binary covariate *Z* as a function of $P(Z=1)$; $\phi$ is the odds ratio of (*X*, *Z*); $\phi = 2.0$

which is a function of the effect of *Z* on the outcome and the extent of the association between *Z* and *X*. Figure 1 plots the limiting bias of the treatment effect estimator under the marginal and partially conditional models as a function of the association between *Z* and *X* and the effect of *Z* (i.e. $\zeta$). The bias increases as the association between *X* and *Z* increases and as the magnitude of $\zeta$ increases. When *X* and *Z* are independent the misspecified marginal model yields consistent estimates of the treatment effect, supporting the use of this method in randomized trials. The partially conditional model, however, yields a biased estimate of treatment effect when an important covariate is omitted even when *X* and *Z* are independent. Thus while the partially conditional model appears to be a more general model than the marginal model, it does not support robust causal inferences about treatment effects in randomized trials when recurrent event follows Poisson processes. It is also apparent from (13) and (16) that the limiting values of the marginal and partially conditional estimators are dependent on the administrative censoring time and the distribution of the random censoring time. We found there to be only a weak dependence on the random censoring rate in both frameworks so we do not report the results of these studies here.

The asymptotic naive and robust standard errors under the misspecified marginal model were also studied using (20) and (22) in Appendix 1, and under the misspecified partially conditional model using (23) and (24) in Appendix 2. Figure 2 plots the trend of asymptotic naive and robust standard errors of the treatment effect as a function of $P(Z=1)$ when $\phi = 2.0$. The robust standard error is larger than the naive standard error under the marginal model with the differences increasing as the effect of the covariate *Z* increases as expected. The robust and naive standard errors are in close agreement under the partially conditional model, in part because the extra-Poisson variation arising from the omission of *Z* is explained by the stratification; Boher and Cook (2006) made a similar observation based on empirical studies. The plots of the asymptotic naive and robust standard errors of the treatment effect estimators have a similar pattern for both the marginal and partially conditional models when $\phi = 1.0$. Similar calculations were carried out for the setting in which *Z*|*X* follows a normal distribution with mean $\theta _0 + \theta _1 X$ and variance $\sigma ^2$; the results are shown for marginal models in Online Resource 1.

## Empirical studies of finite sample behaviour 

Here we consider an empirical study to investigate the finite sample properties of estimators of the treatment effect under the misspecified marginal and partially conditional rate-based models. In Sect. 4.1 we consider the events as generated by a Poisson process and in Sect. 4.2 we consider the case where the events are generated according to a Markov model. In both settings we examine the finite sample properties of estimators from marginal and partially conditional rate-based models in which an important covariate is omitted.

### Misspecified rate-based models for Poisson processes 

For the setting where events are generated by a Poisson process we use the same illustrative setting as in Sect. 3.2. We let $\phi = 0.5, 1.0, 2.0$ and 4.0 to reflect varying strengths of the association between *X* and *Z* when *Z* is binary, and let $P(Z=1) = 0.25$ or 0.50. The effect of *Z* on the event process is set to be $\zeta = 0$, $\log 1.5$ or $\log 3.0$ to reflect no effect to a strong effect. The other parameter settings are the same as those in Sect. 3.2. We generated one thousand samples of size $n=1000$ each. We adopt the marginal and partially conditional models with a single covariate reflecting the treatment, and investigate the empirical properties of the estimators under those misspecified models; see Table 1.

**Table 1:** Empirical frequency of estimates of treatment effect, when omitting covariate *Z* in the assumed rate function under the marginal and partially conditional rate-based models for the recurrent event following a Poisson process; *X* and *Z* are binary correlated with odds ratio $\phi$; $n=1000$ and $nsim=1000$; all numbers for BIAS, ESE, ASE and ECP ($\times 100$) in the table

$\phi$	$\zeta = 0$						$\zeta = \log 1.5$						$\zeta = \log 3.0$					
	BIAS	ESE	ASE$^{1}$	ASE$^2$	ECP$^1$	ECP$^2$	BIAS	ESE	ASE$^{1}$	ASE$^2$	ECP$^1$	ECP$^2$	BIAS	ESE	ASE$^{1}$	ASE$^2$	ECP$^1$	ECP$^2$
Marginal model, $P(Z=1) = 0.25$																		
0.5	$-$ 0.15	5.06	5.14	5.14	96.0	96.1	$-$ 5.59	4.97	4.85	5.01	78.3	80.2	$-$ 17.19	5.72	4.24	5.65	6.6	14.4
1.0	$-$ 0.15	5.06	5.14	5.14	96.0	96.1	$-$ 0.47	5.08	4.85	5.00	93.4	94.2	$-$ 0.09	6.10	4.20	5.65	82.1	92.4
2.0	$-$ 0.15	5.06	5.14	5.14	96.0	96.1	5.74	4.92	4.84	4.99	76.3	78.9	17.22	5.72	4.19	5.61	5.8	14.8
4.0	$-$ 0.15	5.06	5.14	5.14	96.0	96.1	11.04	5.24	4.83	4.97	37.0	38.8	32.51	5.58	4.21	5.53	0.0	0.0
Marginal model, $P(Z=1) = 0.50$																		
0.5	$-$ 0.15	5.06	5.14	5.14	96.0	96.1	$-$ 6.77	4.81	4.61	4.78	68.2	70.1	$-$ 17.28	4.81	3.67	4.92	2.1	5.7
1.0	$-$ 0.15	5.06	5.14	5.14	96.0	96.1	$-$ 0.05	4.55	4.60	4.78	94.9	95.4	0.13	5.06	3.63	4.90	83.7	95.0
2.0	$-$ 0.15	5.06	5.14	5.14	96.0	96.1	7.23	4.85	4.59	4.76	64.3	65.9	17.03	5.09	3.62	4.89	2.9	7.2
4.0	$-$ 0.15	5.06	5.14	5.14	96.0	96.1	13.32	4.75	4.59	4.75	18.2	19.7	33.51	4.75	3.64	4.87	0.0	0.0
Partially conditional model, $P(Z=1) = 0.25$																		
0.5	$-$ 0.15	5.12	5.20	5.19	96.2	96.2	$-$ 4.66	4.95	4.93	4.92	85.2	84.9	$-$ 5.15	4.37	4.37	4.39	79.2	79.3
1.0	$-$ 0.15	5.12	5.20	5.19	96.2	96.2	0.87	4.90	4.90	4.89	94.8	94.7	7.47	4.39	4.27	4.25	57.8	57.6
2.0	$-$ 0.15	5.12	5.20	5.19	96.2	96.2	6.42	4.96	4.88	4.88	72.3	72.4	19.73	3.98	4.22	4.19	0.2	0.2
4.0	$-$ 0.15	5.12	5.20	5.19	96.2	96.2	10.96	5.11	4.87	4.87	40.7	40.6	31.33	4.26	4.24	4.23	0.0	0.0
Partially conditional model, $P(Z=1) = 0.50$																		
0.5	$-$ 0.15	5.12	5.20	5.19	96.2	96.2	$-$ 5.39	4.58	4.69	4.69	80.5	80.1	$-$ 4.96	3.90	3.79	3.82	72.6	73.2
1.0	$-$ 0.15	5.12	5.20	5.19	96.2	96.2	1.21	4.61	4.66	4.65	94.6	94.6	7.47	3.70	3.70	3.65	48.1	47.4
2.0	$-$ 0.15	5.12	5.20	5.19	96.2	96.2	7.70	4.66	4.63	4.61	59.7	59.1	19.78	3.67	3.66	3.60	0.0	0.0
4.0	$-$ 0.15	5.12	5.20	5.19	96.2	96.2	13.84	4.32	4.62	4.60	13.6	13.3	31.77	3.74	3.68	3.69	0.0	0.0

ASE$^1$ and ASE$^2$ are the average of naive standard error and robust standard error, respectively; ECP$^1$ and ECP$^2$ are the empirical coverage probabilities of nominal 95% confidence interval ($\times 100)$ based on naive and robust standard errors, respectively

We find that when *X* and *Z* are independent there is negligible empirical bias of the estimated treatment effect under the marginal model, supporting theory that marginal model is robust and so yields a consistent estimators of the treatment effect in clinical trials. Furthermore, the average robust standard error is in close agreement with the empirical standard error of the estimates in general, while the average naive standard error underestimates the variability, especially when the effect of covariate *Z* is larger supporting the the need for robust standard errors. This can also be seen by comparing the empirical coverage probabilities of nominal 95% confidence interval for ${\hat{\beta }}$ based on naive and robust standard errors. Furthermore, when *X* and *Z* are independent, unlike the marginal model, the partially conditional model yields biased estimates of the treatment effect; this empirical bias is larger when the effect of *Z* on the event process increases. This means that the benefit of randomization is lost when we fit partially conditional models without addressing other covariate effects.

When *X* and *Z* are not independent, there is significant bias of the estimates for treatment effect under both models, and the bias increases when the association between *X* and *Z* is stronger or the effect of the omitted *Z* on the event process becomes larger. These findings agree with our theoretical results in Sect. 3.2. Note that under the misspecified marginal model, the robust standard errors accurately reflect the empirical variation indicating that they provide protection from the misspecification to some extent. Due to the significantly large bias of the estimates of treatment effect under the misspecified model, the empirical coverage probabilities of the 95% confidence intervals are unacceptably low when *X* and *Z* are correlated. We also note that under the misspecified partially conditional model, there is reasonable agreement between the average model-based standard errors and the average robust standard errors; this is in alignment with the theoretical results of Sect. 3.2. The results of additional simulation studies involving normally distributed *Z* lead to similar conclusions; see Online Resource 2 for results.

### Misspecified rate-based models for Markov processes 

**Fig. 3:**
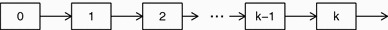
Multistate representation of a recurrent event process

We now consider the setting in which the events are generated by a progressive multistate process with states labeled $0, 1, \ldots$ representing the cumulative number of events and $\{N_i(t), 0\le t\}$ the event process as before; the state-space diagram is depicted in Figure 3. We assume that $k \rightarrow k+1$ transitions occur according to a Markov intensity

$$\begin{aligned} \lim _{\Delta t \downarrow 0} \frac{P(N_i (t+\Delta t^{-}) = k+1|N_{i}(t^{-}) = k, X_i, Z_i)}{\Delta t} = q_k (t) \exp (\eta X_i + \zeta Z_i ), \end{aligned}$$

where $q_k(t)$ is a baseline transition rate, $k=0, 1, \ldots$. We let $q_{k+1} (t) = q_k (t)e^{\alpha }$, $k=0, \ldots , K$ so that the occurrence of each event increases the baseline rate of the next event up until the $(K+1)$st event and set $q_{k+1}(t) = q_k (t)$ for $k=K+1, \ldots , K_m$ so that the risk does not increase beyond the $(K+1)$st event; data are generated for at most $K_m$ transition times but this is chosen to be large enough that the probability of entering the absorbing state over the planned period of observation is essentially zero.

Time-homogeneous transition intensities are obtained by letting $q_0 (t) =q_0$. We let *Q* denote the $(K_m + 1) \times (K_m + 1)$ transition intensity matrix with $Q_{jj} = -q_{j-1}$ entries on the diagonal $Q_{j, j+1} = q_{j-1}$ above the diagonal and $Q_{jl} = 0$ for $l \ne j$ or $j+1$; $j=1, 2, \ldots , K_m + 1$. The Chapman–Kolmogorov equations then give,

18 $$\begin{aligned} P(s, s+t|X = 0, Z=0) = \exp (Q t), \end{aligned}$$

where $P(s, s+t|X=0, Z=0) = P(0, t|X=0, Z=0)$ and $P_{jl}(0, t|X=0, Z=0) = P(Z(t) = l |Z(0) = j, X=0, Z=0)$ (Cox and Miller 1965).

As before we consider the case when *Z* is Bernoulli with $P(Z=1) = p_z$ and the odds ratio for the association between *X* and *Z* is $\phi$. We let $\alpha =\log 1.05$ so there is a 5% increase in the risk of an event each time an event occurs up to $K=5$, and let $K_m = 20$. We determine $q_0$ so that $\mu (1|X=0, Z=0) = 2$ where

19 $$\begin{aligned} \mu (t|X=0, Z=0) = \sum _{k=0}^{K_m} k \cdot P(Z(t) = k|Z(0)=0, X=0, Z=0) \end{aligned}$$

is the expected number of events at time t given $X=Z=0$; the other parameter settings are the same as in Sect. 4.1. We generate one thousand samples of size $n=1000$ each from this Markov process. The marginal and partially conditional models are fitted with only a treatment indicator, and the empirical properties of the resulting estimators are summarized in Table 2.

**Table 2:** Empirical frequency of estimates of treatment effect, when omitting covariate *Z* in the assumed rate function under the marginal and partially conditional rate-based models for the recurrent event following a Markov process; *X* and *Z* are binary correlated with odds ratio $\phi$; $n=1000$ and $nsim=1000$; all numbers for BIAS, ESE, ASE and ECP ($\times 100$) in the table; $\alpha = \log (1.05)$

$\phi$	$\zeta = 0$						$\zeta = \log 1.5$						$\zeta = \log 3.0$					
	BIAS	ESE	ASE$^{1}$	ASE$^2$	ECP$^1$	ECP$^2$	BIAS	ESE	ASE$^{1}$	ASE$^2$	ECP$^1$	ECP$^2$	BIAS	ESE	ASE$^{1}$	ASE$^2$	ECP$^1$	ECP$^2$
Marginal model, $P(Z=1) = 0.25$																		
0.5	$-$ 1.40	5.24	5.13	5.34	93.4	94.7	$-$ 7.30	5.15	4.83	5.24	67.3	72.8	$-$ 20.70	6.26	4.15	6.01	2.5	8.7
1.0	$-$ 1.40	5.24	5.13	5.34	93.4	94.7	$-$ 1.43	5.17	4.82	5.22	92.3	94.9	$-$ 2.19	6.02	4.10	6.03	78.5	93.3
2.0	$-$ 1.40	5.24	5.13	5.34	93.4	94.7	4.58	5.11	4.81	5.21	82.4	85.6	16.14	6.03	4.09	5.98	10.0	23.8
4.0	$-$ 1.40	5.24	5.13	5.34	93.4	94.7	9.98	5.24	4.81	5.20	45.9	51.6	33.26	5.83	4.12	5.88	0.0	0.0
Marginal model, $P(Z=1) = 0.50$																		
0.5	$-$ 1.40	5.24	5.13	5.34	93.4	94.7	$-$ 9.28	5.03	4.58	5.01	46.9	54.2	$-$ 20.59	5.28	3.56	5.20	0.8	2.1
1.0	$-$ 1.40	5.24	5.13	5.34	93.4	94.7	$-$ 1.56	4.86	4.56	5.00	92.0	95.0	$-$ 2.47	5.19	3.52	5.19	77.5	91.9
2.0	$-$ 1.40	5.24	5.13	5.34	93.4	94.7	5.68	5.00	4.55	4.99	72.6	77.9	15.56	5.21	3.51	5.19	5.0	14.9
4.0	$-$ 1.40	5.24	5.13	5.34	93.4	94.7	12.57	4.94	4.55	4.98	22.7	28.0	32.87	5.41	3.53	5.17	0.0	0.0
Partially conditional model, $P(Z=1) = 0.25$																		
0.5	$-$ 0.21	5.06	5.19	5.17	95.4	95.6	$-$ 4.46	4.84	4.91	4.90	86.2	86.0	$-$ 5.06	4.31	4.28	4.32	79.2	79.9
1.0	$-$ 0.21	5.06	5.19	5.17	95.4	95.6	0.94	4.82	4.88	4.87	94.4	94.3	7.63	4.23	4.17	4.15	55.5	54.7
2.0	$-$ 0.21	5.06	5.19	5.17	95.4	95.6	6.41	4.78	4.85	4.85	73.6	73.5	19.58	4.16	4.12	4.10	0.2	0.2
4.0	$-$ 0.21	5.06	5.19	5.17	95.4	95.6	11.33	4.87	4.85	4.84	35.9	35.9	31.17	4.21	4.14	4.17	0.0	0.0
Partially conditional model, $P(Z=1) = 0.50$																		
0.5	$-$ 0.21	5.06	5.19	5.17	95.4	95.6	$-$ 5.94	4.69	4.66	4.66	75.4	74.7	$-$ 4.83	3.72	3.68	3.72	74.3	75.6
1.0	$-$ 0.21	5.06	5.19	5.17	95.4	95.6	1.13	4.51	4.62	4.61	94.5	94.5	7.45	3.49	3.58	3.53	44.9	44.2
2.0	$-$ 0.21	5.06	5.19	5.17	95.4	95.6	7.73	4.61	4.59	4.58	59.8	59.3	19.01	3.56	3.54	3.49	0.0	0.0
4.0	$-$ 0.21	5.06	5.19	5.17	95.4	95.6	14.00	4.55	4.58	4.56	14.1	14.3	30.43	3.75	3.55	3.58	0.0	0.0

ASE$^1$ and ASE$^2$ are the average of naive standard error and robust standard error, respectively; ECP$^1$ and ECP$^2$ are the empirical coverage probabilities of nominal 95% confidence interval ($\times 100)$ based on naive and robust standard errors, respectively

The partially conditional model is the correct model when $\zeta = 0$ and yields consistent estimators of treatment effect; see the column of results headed $\zeta =0$ in Table 2. Although the marginal model ignores the state-dependent transition intensity, statistical inference for the treatment effect remains valid if the robust standard error is used when $\zeta =0$. When $\zeta \ne 0$ however, both the marginal and partially conditional models omitting *Z* are misspecified; here the resulting estimators are biased and the confidence intervals have poor empirical coverage probability. When $X \perp Z$ and $\zeta = \log 1.5$, the marginal and partially conditional models yield valid inferences. This does not hold with larger $\alpha$ or when the covariate *Z* is normally distributed (see Online Resource 2 for more simulation results). Therefore, our empirical studies suggest that when the true model is Markov, ignoring the important confounders or even independent prognostic variables (i.e. $X \perp Z$ at $t=0$) can yield estimators of treatment effect which are susceptible to misspecification. Whether valid estimates of the treatment effect can be obtained in the clinical trial setting under these two models therefore depends on how large the effect of omitted prognostic variables are as well as their distribution. This can be re-expressed by stating that inferences based on partially conditional rate-based analysis are sensitive to departures from the Markov assumption on which it is formally justified. Model assessment has a particularly useful role here and simulations and sensitivity analyses may be worthwhile to investigate the impact of model violations on the performance of estimators and tests based on marginal or partially conditional models.

## Application to a trial in cystic fibrosis 

Cystic fibrosis is a respiratory disease with airway obstruction caused by the accumulation of mucus in the lungs due to extracellular DNA; this results in recurrent pulmonary exacerbations. When delivered to the lungs in an aerosolized form, a highly purified recombinant form of DNase I called rhDNase cuts extracellular DNA, reducing the viscoelasticity of airway secretions and improving clearance. In a randomized double-blind trial 321 individuals were assigned to receive rhDNase and 324 we assigned to a placebo treatment (Fuchs et al. 1994). The primary purpose of this study was to investigate the effect of rhDNase on the suppression of exacerbations so to this end the onset times of exacerbations were recorded over the study period of approximately 169 days. In the control arm 139 individuals had at least one exacerbation, 42 had at least two exacerbations, and 18 had at least three exacerbations; in the rhDNase arm these numbers were 104, 39 and 9 respectively. The baseline forced expiratory volume (FEV) is a measure of lung function known to be highly associated with the onset of exacerbations; it was centered in the analyses that follow by subtracting the mean value and we denote it by FEVC. The data are available at the website for Cook and Lawless (2007).

**Table 3:** Estimates of treatment effect for cystic fibrosis trial using marginal and partially conditional models with four strata based on no events, 1 event, 2 events and $\ge 3$ events when ignoring or controlling for the centered forced expiratory volume (FEVC)

	$\beta$	$\exp (\beta )$	Robust S.E.	*P* value
Marginal model				
Without FEVC	$-$ 0.271	0.763	0.124	0.029
With FEVC	$-$ 0.267	0.766	0.120	0.027
Partially conditional model				
Without FEVC	$-$ 0.234	0.791	0.108	0.030
With FEVC	$-$ 0.246	0.782	0.109	0.024

We fit the marginal and partially conditional models with the treatment indicator alone, and when controlling for the baseline FEVC. Since only a few individuals experienced more than 3 events, four time-dependent strata were defined based on no events ($N_i(t^-)=0$), 1 event ($N_i(t^-)=1$), 2 events ($N_i(t^-)=2$), and $\ge 3$ events ($N_i(t^-) \ge 3$). The results summarized in Table 3 reveal that the estimates and conclusions are comparable across the four analyses, but we make comments here related to the findings of the theory and empirical studies of Sections 3 and 4. First there is very close agreement between the estimates of treatment effect from the marginal analysis whether FEVC is controlled for or not—this is to be expected based on the results in Sect. 3.2. There is a slightly smaller standard error for the coefficient in the adjusted analysis as FEVC explains some of the variation in the event risk across individuals. The estimate of the treatment effect is smaller from the partially conditional (stratified) analyses decreasing from $-0.271$ to $-0.234$ in the models not adjusting for FEVC for example. This reduction in the estimated treatment effect is accompanied by a reduction in the robust standard error in the partially conditional analysis, and as a result the *p* values are virtually identical at 0.029 and 0.030 for the Wald tests. Similar findings are observed when controlling for FEVC.

**Fig. 4:**
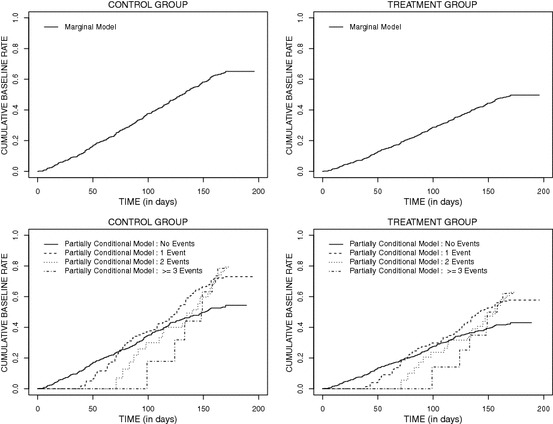
Estimated cumulative marginal rate functions (top row) and cumulative stratified rate functions (bottom row) for the cystic fibrosis trial

For completeness we plot the semiparametric estimates of the cumulative baseline rates under the marginal (top row) and partially conditional baseline rates (bottom row) in Figure 4. While the plots are provided for each treatment group they are obtained from one fitted model for each analysis. The effect of treatment is evident graphically from the lower slope of the estimate in the rhDNase arm (top row). Moreover the estimates of the cumulative stratified baseline transition rates show the increased risk of event occurrence with each event; this is inferred by the progressively steeper estimates reflecting higher risks at any time.

Motivated by the suggestions we provide in Sect. 4.2, we carry out a small simulation study to mimic the cystic fibrosis data and investigate the behaviour of the estimates under the marginal and partially conditional models. Based on Figure 4, we assume the recurrent event follows a Markov process as we specified in Sect. 4.2 with $K=2$ and $K_m = 20$. We fit the model $\lambda _i(t) = q_0 \exp (\eta X_i + \zeta Z_i + \alpha N_i(t^-))$ and obtain the estimates ${\hat{\eta }} = -0.228$, ${\hat{\zeta }}=-0.015$ and ${\hat{\alpha }}=0.343$. The Nelson–Aalen estimates of the cumulative baseline intensities could be obtained with the slopes providing a way of selecting $q_0$; here we take $q_0=0.0032$. The centered baseline forced expiratory volume approximately follows a normal distribution with mean 0 and standard deviation 26. In this simulation study we took the covariate *Z* to follow a similar distribution as FEVC, which is normally distributed with mean 0 and standard deviation $\sigma _z = 20$, 26, or 30; we study settings with slightly lower and slightly higher variability. We let the effect of FEVC on the event rate be $\zeta = -0.50$, -0.10, -0.01, 0.00, and 0.20. Using these values, we could generate the event times for $n=645$ individuals. The empirical frequency of estimates under the marginal and partially conditional models with only the treatment indicator are summarized in Table 4. We note that when there is no effect of *Z* on the event process, the partially conditional model with only treatment indicator is the correct model and hence leads to consistent estimation of the treatment effect. Although the marginal model ignores the state-dependent transition intensity, statistical inference for the treatment effect is still valid when robust variance estimates are used. When $\zeta \ne 0$, both the marginal and partially conditional models omitting *Z* result in biased estimates and the confidence intervals have poor empirical coverage probability.

**Table 4:** Empirical frequency of estimate for treatment effect, when omitting covariate *Z* in the assumed rate function under the marginal and partially conditional models for the recurrent event following a Markov process; *Z* is normally distributed with mean 0 and standard deviation $\sigma _z$, and *X* and *Z* are independent; $n=645$ and $nsim=1000$; all numbers for BIAS, ESE, ASE and ECP ($\times 100$) in the table

$\zeta$	Marginal model						Partially conditional model					
	BIAS	ESE	ASE$^{1}$	ASE$^2$	ECP$^1$	ECP$^2$	BIAS	ESE	ASE$^{1}$	ASE$^2$	ECP$^1$	ECP$^2$
$$\sigma _z = 20$$												
0.20	14.47	13.19	3.22	12.89	19.8	79.6	21.61	5.01	3.27	4.92	0.2	0.8
0.00	$-$ 3.29	12.14	11.00	12.22	92.2	94.7	$-$ 0.68	10.94	11.06	11.03	96.1	96.1
$-$ 0.01	$-$ 3.00	12.11	10.84	12.27	91.4	94.5	0.02	10.69	10.90	10.88	95.0	94.8
$-$ 0.10	6.34	15.41	4.41	15.46	37.0	93.2	18.47	6.96	4.46	6.93	9.0	25.0
$-$ 0.50	20.40	12.15	2.74	11.70	8.3	59.9	22.52	4.36	2.83	4.12	0.0	0.0
$$\sigma _z = 26$$												
0.20	16.21	12.30	3.01	12.36	17.6	74.1	21.79	4.83	3.07	4.57	0.0	0.7
0.00	$-$ 3.29	12.14	11.00	12.22	92.2	94.7	$-$ 0.68	10.94	11.06	11.03	96.1	96.1
$-$ 0.01	$-$ 3.46	12.54	10.73	12.32	89.8	94.0	$-$ 0.03	10.96	10.80	10.77	94.7	94.6
$-$ 0.10	10.22	14.12	3.80	14.26	30.4	89.9	20.00	6.14	3.85	5.91	2.3	9.5
$-$ 0.50	21.00	11.71	2.68	11.57	8.8	54.6	22.74	4.21	2.80	4.04	0.0	0.0
$$\sigma _z = 30$$												
0.20	18.56	12.25	2.93	12.17	13.7	66.3	22.19	4.62	3.00	4.44	0.0	0.3
0.00	$-$ 3.29	12.14	11.00	12.22	92.2	94.7	$-$ 0.68	10.94	11.06	11.03	96.1	96.1
$-$ 0.01	$-$ 2.69	12.50	10.63	12.38	89.5	93.6	0.88	10.82	10.70	10.67	94.8	94.9
$-$ 0.10	12.40	13.62	3.56	13.72	28.4	85.6	20.95	5.58	3.61	5.50	0.8	5.1
$-$ 0.50	21.40	11.25	2.65	11.48	7.1	53.9	22.42	4.29	2.78	4.01	0.0	0.0

## Discussion

Marginal and partially conditional semiparametric models have received considerable attention in recent years as methods for assessing the effect of therapeutic interventions on the basis of recurrent events. The marginal rate-based model is viewed as offering a robust approach to assessing treatment effects but it is susceptible to the effects of model misspecification; while we have demonstrated this when the true event generating process is Markov, this arises whenever the basic multiplicative assumption of covariate effects is not satisfied. While the partially conditional model represents a generalization of the marginal model through the introduction of time-dependent strata, the strata are defined based on the cumulative number of events which is responsive to treatment and other risk factors which also having effect on the outcome. Conditioning on time-dependent variables which are realized post-randomization and potentially responsive to treatment has been known to be problematic for some time (Kalbfleisch and Prentice 2002). Hernán (2010) points out that analyses based on Cox regression models incorporate such conditioning implicitly through the comparison of covariate distributions among those individuals who are uncensored and event-free at each failure time post-randomization; see also Aalen et al. (2015). Here we investigate in detail the implications of conditioning on the cumulative number of events in a partially conditional model for recurrent event analyses. The findings mean that the full marginal model should be used in randomized trials since, as demonstrated here, it can yield an estimate of treatment effect with a simple causal interpretation. Careful examination of the multiplicative assumption is warranted however to ensure the assumption is reasonable.

### Electronic supplementary material

Below is the link to the electronic supplementary material.

Supplementary material 1 (pdf 260 KB)

## Appendix 1: Asymptotic naive and robust variance of treatment effect estimate under a misspecified marginal model

Under a misspecified marginal model where a covariate *Z* is omitted from the rate function, the asymptotic properties of the estimator for the treatment effect are given in Sect. 3. Here we derive the explicit forms of ${{\mathcal {A}}}(\beta )$ and ${{\mathcal {B}}}(\beta )$ used to calculate the asymptotic model-based and robust variances of the estimator. Using the notation of the paper,

$$\begin{aligned} {{\mathcal {A}}}(\beta )= & {} E\left[ - \partial U_i (\beta ) / \partial \beta \right] = E\left[ \int _0^{\infty } Y_i (t)\cdot \left\{ \frac{s^{(2)}(\beta , t)}{s^{(0)}(\beta , t)} - \left( \frac{s^{(1)}(\beta , t)}{s^{(0)}(\beta , t)}\right) ^2 \right\} \cdot dN_i (t) \right] \\= & {} E_{Y_i (t), X_i, Z_i}\left[ \int _0^{\infty } Y_i (t) \cdot \left\{ \frac{s^{(2)}(\beta , t)}{s^{(0)}(\beta , t)} - \left( \frac{s^{(1)}(\beta , t)}{s^{(0)}(\beta , t)}\right) ^2 \right\} \cdot e^{\eta X_{i} + \zeta Z_{i}}\cdot d\mu _0(t) \right] \\= & {} \int _0^{A} {{\mathcal {G}}}(t) \cdot \left\{ \frac{s^{(2)}(\beta , t)}{s^{(0)}(\beta , t)} - \left( \frac{s^{(1)}(\beta , t)}{s^{(0)}(\beta , t)}\right) ^2 \right\} \cdot E\left( e^{\eta X_{i} + \zeta Z_{i} }\right) \cdot d\mu _0(t), \end{aligned}$$

where $s^{(2)}(\beta , t) = n^{-1}\sum _{i=1}^{n}E[Y_i (t) e^{\beta X_{i}}X_{i}^2] = {{\mathcal {G}}}(t) E[e^{\beta X_{i} }X_{i}^2] = s^{(1)}(\beta , t)$ for a binary treatment covariate $X_i$. Note that

$$\begin{aligned} \frac{s^{(2)}(\beta , t)}{s^{(0)}(\beta , t)} = \frac{s^{(1)}(\beta , t)}{s^{(0)}(\beta , t)} = \frac{\exp (\beta ) P(X_i = 1)}{\exp (\beta ) P(X_i = 1) + P(X_i = 0)} . \end{aligned}$$

We define $\Delta (\beta ) = s^{(r)}(\beta , t)/s^{(0)}(\beta , t)$, $r=1, 2$, to write

20 $$\begin{aligned} {{\mathcal {A}}}(\beta )= & {} (\Delta (\beta ) - \Delta ^2 (\beta ) )\cdot E\left( e^{\eta X_{i} + \zeta Z_{i}}\right) \cdot \int _0^{A} {{\mathcal {G}}}(t) \cdot d\mu _0(t). \end{aligned}$$

Since $n^{-1/2} U(\beta )$ is asymptotically equivalent to $n^{-1/2} \sum _{i=1}^{n} w_i (\beta )$ (Lin and Wei 1989), asymptotically $n^{-1/2} U(\beta ) \sim \text{ N }(0, {{\mathcal {B}}}(\beta ))$, where ${{\mathcal {B}}}(\beta ) = E\left( w_i (\beta )w'_i (\beta )\right)$, and

21 $$\begin{aligned} w_i (\beta ) = \int _0^{\infty } Y_i (t) \left( X_i - \frac{s^{(1)}(\beta , t)}{s^{(0)}(\beta , t)}\right) \left( dN_i(t) - \frac{\exp (\beta X_i)}{s^{(0)}(\beta , t)} d{\bar{F}}(t)\right) . \end{aligned}$$

Following the same strategy, we derived the explicit form of ${{\mathcal {B}}}(\beta )$ under the misspecified marginal model in Online Resource 3, and obtained that

22 $$\begin{aligned} {{\mathcal {B}}} (\beta )= & {} \left( \int _0^A {{\mathcal {G}}}(t)d\mu _0(t)\right) \cdot E\left[ (X_i - \Delta (\beta ))^2 e^{\eta X_i + \zeta Z_i}\right] \nonumber \\&+ ~ Q\times \Bigg [E\left\{ \left( X_i - \Delta (\beta )\right) ^2 \cdot e^{2 (\eta X_i + \zeta Z_i)}\right\} \nonumber \\&-\, 2\times E\left\{ \left( X_i - \Delta (\beta )\right) ^2 \cdot e^{\beta X_i + \eta X_i+ \zeta Z_i}\right\} \cdot \frac{E[e^{\eta X_i + \zeta Z_i}]}{E[e^{\beta X_i}]} \nonumber \\&+~ E\left\{ \left( X_i - \Delta (\beta )\right) ^2 \cdot e^{2 \beta X_i}\right\} \left( \frac{E[e^{\eta X_i + \zeta Z_i}]}{E[e^{\beta X_i}]}\right) ^2\Bigg ], \end{aligned}$$

where $$Q = 2 \int \limits _0^{A}{{\mathcal {G}}}(s) \mu _0 (s) d\mu _0 (s)$$.

Through (20) and (22) we can now obtain the asymptotic naive and robust variance of estimate for treatment effect under the misspecified marginal model when the recurrent event follows Poisson processes. If *Z* has no effect on the outcome (i.e. $\zeta = 0$) then ${{\mathcal {A}}}(\beta ) = {{\mathcal {B}}}(\beta )$, which means when the marginal model is correctly specified, then the naive variance and robust variance of the treatment effect estimate are the same.

## Appendix 2: Asymptotic naive and robust variance of treatment effect estimate under misspecified partially conditional model

Under the misspecified partially conditional model where covariate *Z* is omitted, the estimating function for $\beta$ is given in Sect. 3 and we have

23 $$\begin{aligned} \tilde{{{\mathcal {A}}}}(\beta )= & {} E\left[ -\partial {\tilde{U}}_i (\beta ) /\partial \beta \right] \nonumber \\= & {} E\left[ \sum _{k=1}^{\infty } \int _0^{\infty } {\bar{Y}}_{ik}(s)\cdot \left\{ \frac{s^{(2)}_k (\beta , s)}{s^{(0)}_k(\beta , s)}-\left( \frac{s^{(1)}_k (\beta , s)}{s^{(0)}_k (\beta , s)}\right) ^2\right\} \cdot dN_{ik}(s)\right] \nonumber \\= & {} E\left[ \sum _{k=1}^{\infty } \int _0^{\infty } {\bar{Y}}_{ik}(s) \cdot \left\{ \frac{s^{(2)}_k (\beta , s)}{s^{(0)}_k(\beta , s)}-\left( \frac{s^{(1)}_k (\beta , s)}{s^{(0)}_k (\beta , s)}\right) ^2\right\} d\mu _i (s)\right] \nonumber \\= & {} \sum _{k=1}^{\infty } \int _0^{\infty } s_k^{(0)}(s) \cdot \left\{ \frac{s^{(2)}_k (\beta , s)}{s^{(0)}_k(\beta , s)}-\left( \frac{s^{(1)}_k (\beta , s)}{s^{(0)}_k (\beta , s)}\right) ^2\right\} ds, \end{aligned}$$

where $s_k^{(r)}(\beta , s) = E[{\bar{Y}}_{ik}(s) e^{\beta X_i} X_i^{\otimes r} ]$, $r=0, 1, 2$, and $s_{k}^{(0)}(s) = E[{\bar{Y}}_{ik}(s) \rho _i (s)]$. Furthermore, since $n^{-1/2} {\tilde{U}}(\beta )$ is asymptotically equivalent to $n^{-1/2}\sum _{i=1}^{n} {\tilde{w}}_{i}(\beta )$, where

$$\begin{aligned} {\tilde{w}}_{i}(\beta )= & {} \sum _{k=1}^{\infty } {\tilde{w}}_{ik}(\beta ) = \sum _{k=1}^{\infty } \int _0^{\infty } {\bar{Y}}_{ik}(s) \left( X_i - \frac{s^{(1)}_k (\beta , s)}{s^{(0)}_k (\beta , s)}\right) \cdot \left( dN_{ik}(s) - \frac{e^{\beta X_i}}{s_{k}^{(0)}(\beta , s)}~d{\bar{F}}_k (s) \right) , \end{aligned}$$

and $d{\bar{F}}_k (s) = E[{\bar{Y}}_{ik}(s)dN_{ik}(s)] = E[{\bar{Y}}_{ik}(s) d\mu _i (s)] = s_k^{(0)}(s) ds$. The asymptotic variance of $n^{-1/2} {\tilde{U}}(\beta )$ is $\tilde{{{\mathcal {B}}}}(\beta ) = E[{\tilde{w}}_i (\beta ) {\tilde{w}}'_i (\beta )]$. In Online Resource 3, we derived the explicit form of $\tilde{{\mathcal {B}}}(\beta )$ under the misspecified partially conditional model, which can be written as

24 $$\begin{aligned} \tilde{{{\mathcal {B}}}}(\beta )= & {} \sum _{j=1}^{\infty } E\left[ \int _0^{A} {{\mathcal {G}}}(t) \cdot P(Y_{ij}(t) = 1) \cdot \left( X_i - \Delta _j (\beta , t)\right) ^{2} d\mu _i (t)\right] \nonumber \\&+\, 2*\sum _{j > k} E\Bigg \{\int \limits _{0}^{A}\int \limits _{0}^{t} {{\mathcal {G}}}(t)\cdot \left( X_i - \Delta _j (\beta , t)\right) \cdot \left( X_i - \Delta _k (\beta , s)\right) \cdot P(Y_{ik}(s) =1)\nonumber \\&\times \left( \rho _i (t) - \frac{e^{\beta X_i}s_{j}^{(0)}(t)}{s_{j}^{(0)}(\beta , t)}\right) \times \Bigg (P(Y_{ij}(t) = 1|dN_{ik}(s) = 1, Y_{ik}(s)=1)\rho _i (s) \nonumber \\&- \frac{e^{\beta X_i}s_{k}^{(0)}(s)}{s_{k}^{(0)}(\beta , s)}P(Y_{ij}(t) = 1|Y_{ik}(s)=1) \Bigg ) ds dt \Bigg \}, \end{aligned}$$

where $\Delta _j (\beta , t) = s_j^{(1)}(\beta , t) / s_j^{(0)}(\beta , t)$. Therefore, we can obtain the naive variance, $\tilde{{{\mathcal {A}}}}^{-1}(\beta )$, and robust variance, $\tilde{{{\mathcal {A}}}}^{-1}(\beta ) \tilde{{{\mathcal {B}}}}(\beta ) [\tilde{{{\mathcal {A}}}}^{-1}(\beta )]'$, of the estimate for treatment effect under the partially conditional model where covariate *Z* is omitted from the model.
