# Supplementary material for: The effect of omitted covariates in marginal and partially conditional recurrent event analyses
Source: Lifetime Data Anal. 2018 May 16;25(2):280–300. doi: 10.1007/s10985-018-9430-y (PMC6423006; doi:10.1007/s10985-018-9430-y)
Supplement: Supplementary file 1 — Supplementary material 1 (pdf 260 KB) [file 10985_2018_9430_MOESM1_ESM.pdf]

Lifetime Data Analysis manuscript No.
(will be inserted by the editor)

Supplementary Material for *The effect of omitted covariates in marginal and partially conditional recurrent event analyses*

Received: date / Accepted: date

Online Resource 1: Limiting behaviour of estimators of treatment effect under misspecified marginal model

We assume the recurrent event follows a Poisson process with a Weibull rate function $\rho(t) = \rho_0(t) \exp(\eta X + \zeta Z)$ with $\rho_0(t) = \lambda\kappa(\lambda t)^{\kappa-1}$. X is a binary treatment indicator and Z is a fixed covariate with $Z|X \sim N(\theta_0 + \theta_1 X, \sigma^2)$. By equation (15), we could calculate the limiting bias of treatment effect estimator under the marginal rate-based model with only treatment indicator. The asymptotic naive and robust standard errors under the misspecified marginal model can also be evaluated using equations (20) and (22) in Appendix 1. We consider the same parameter settings as in Sect. 3.2 but with conditionally normally distributed confounder Z. Figure S1.1 shows the limiting bias of estimates of treatment effect under the marginal model when omitting the potential confounder Z and the asymptotic naive and robust standard errors of the corresponding estimators are shown in Figure S1.2

We can find that when X and Z are independent, the misspecified marginal model can still yield consistent estimate of the treatment effect. When X and Z are correlated, the limiting bias increases as the association between X and Z increases and as the magnitude of ζ increases. But the variability of Z has little effect on the limiting bias. Furthermore, under the misspecified marginal model, the robust standard error is larger than the naive standard error with the differences increasing when the effect of the covariate Z increase and as the variability of Z increases.

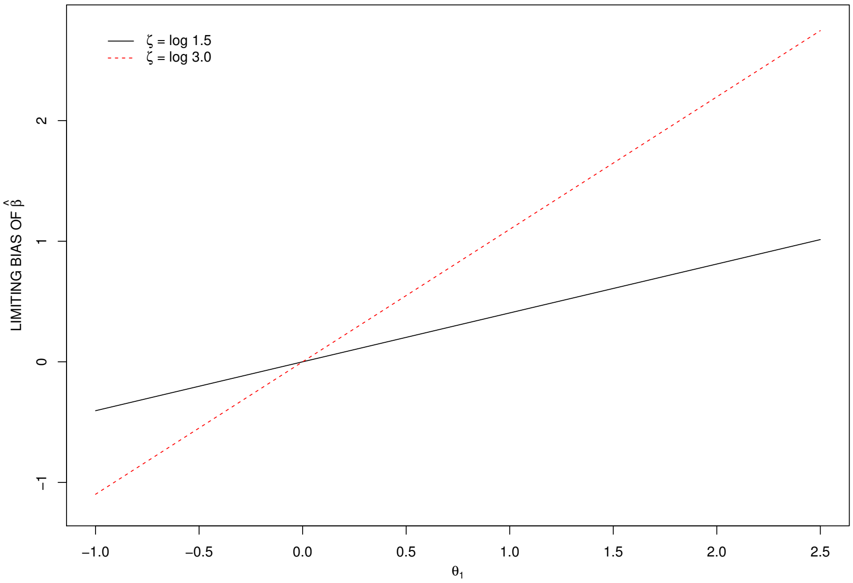

Fig. S1.1 Limiting bias of estimates of treatment effect under a marginal model when omitting the covariate Z; $Z|X$ is normally distributed with mean $1 + \theta_1 X$ and variance 0.5.

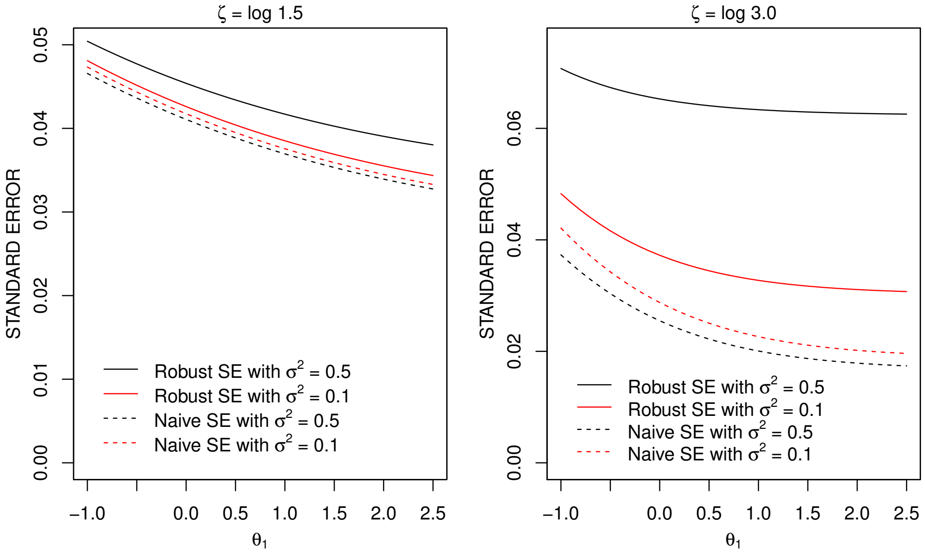

Fig. S1.2 Asymptotic naive and robust standard errors of estimates of treatment effect under the marginal model omitting a conditional normal covariate Z as a function of θ_1; $Z|X$ follows a normal distribution with mean $1 + \theta_1 X$ and variance σ^2.

Online Resource 2: Empirical studies of finite sample behaviour under misspecified rate-based models

2.1 Recurrent event follows a Poisson process

Here we consider a similar simulation study as in Sect. 4.1 to investigate the finite sample properties of estimators of the treatment effect under the marginal and partially conditional models when omitting a normally distributed confounder Z, where $Z|X \sim N(\theta_0 + \theta_1 X, \sigma^2)$. The recurrent event is a Poisson process with a Weibull rate function $\rho(t) = \lambda\kappa(\lambda t)^{\kappa-1}\exp(\eta X + \zeta Z)$. We let $\theta_0 = 1$, and $\theta_1 = 0.0$, 0.5 or 2.0 to reflect the varying strengths of the association between X and Z. The variability of Z is set to be $\sigma^2 = 0.1$ or 0.5. We consider $\zeta = 0$, $\log 1.5$, or $\log 3.0$ to reflect the none to strong effect of covariate Z on the event process. The other parameter settings are same as in Sect. 4.1. The marginal and partially conditional models with a single treatment indicator are adopted and the empirical properties of the estimators are summarized in Table S2.1.

We find that when there is no effect of Z on the event process, both marginal and partially conditional models yield consistent estimates of treatment effect. When X and Z are independent there is negligible empirical bias of the estimated treatment effect under the marginal model, while the partially conditional model yields biased estimates. This empirical bias is larger when the effect of Z on the event process increases. This means the marginal model is robust in clinical trials but the benefit of randomization is lost when we fit partially conditional model without addressing other covariates effect. Furthermore the robust standard error is in close agreement with the empirical standard error in general, while the average naive standard error underestimates the variability under the misspecified marginal model. When X and Z are not independent, there is significant bias of the estimates of treatment effect under both models, and the bias increases when the association between X and Z is stronger or the effect of Z on the event process becomes larger.

2.2: Recurrent event follows a Markov process

We now consider the same Markov process as in Sect. 4.2 to govern the recurrent event. The potential confounder Z is conditionally normally distributed here, and other parameter settings are same as in Sect. 4.2. The marginal and partially conditional models with only a single treatment indicator are fitted, and the empirical properties of the resulting estimators are summarized in Table S2.2. The partially conditional model is the correct model when $\zeta = 0$ and leads to consistent estimator of treatment effect. The marginal model can still lead to valid inference when $\zeta = 0$ if the robust standard error is used. When $\zeta \neq 0$, however, both the marginal and partially conditional models omitting Z result in biased estimators of the treatment effect.

Table S2.1 Empirical frequency of estimate for treatment effect when omitting covariate Z in the assumed marginal or partially conditional rate functions for the recurrent event following a Poisson process; $Z|X \sim N(1+\theta_1 X, \sigma^2)$; $n = 1000$ and $nsim = 1000$; all numbers for BIAS, ESE, ASE and ECP (×100) in the table.

	$\zeta = 0$						$\zeta = \log 1.5$						$\zeta = \log 3.0$					
θ_1	BIAS	ESE	ASE^1	ASE^2	ECP^1	ECP^2	BIAS	ESE	ASE^1	ASE^2	ECP^1	ECP^2	BIAS	ESE	ASE^1	ASE^2	ECP^1	ECP^2
Marginal Model, $\sigma^2 = 0.1$																		
0.0	-0.03	5.14	5.14	5.13	95.7	95.6	-0.24	4.40	4.18	4.26	93.9	94.3	0.04	3.81	2.88	3.72	86.7	94.8
0.5	-0.03	5.14	5.14	5.13	95.7	95.6	20.44	4.14	3.95	4.03	0.1	0.2	54.96	3.43	2.51	3.44	0.0	0.0
2.0	-0.03	5.14	5.14	5.13	95.7	95.6	80.96	3.47	3.45	3.55	0.0	0.0	219.77	3.11	2.02	3.11	0.0	0.0
Marginal Model, $\sigma^2 = 0.5$																		
0.0	-0.03	5.14	5.14	5.13	95.7	95.6	-0.09	4.55	4.11	4.53	92.2	95.0	0.21	6.51	2.55	6.47	56.0	94.6
0.5	-0.03	5.14	5.14	5.13	95.7	95.6	20.09	4.36	3.89	4.34	0.1	0.2	55.31	6.32	2.22	6.39	0.0	0.0
2.0	-0.03	5.14	5.14	5.13	95.7	95.6	80.92	3.95	3.40	3.91	0.0	0.0	219.78	6.29	1.79	6.23	0.0	0.0
Partially Conditional Model, $\sigma^2 = 0.1$																		
0.0	-0.06	5.19	5.19	5.18	95.2	95.5	0.35	4.42	4.24	4.23	93.5	93.6	6.64	3.06	2.96	2.97	38.3	38.1
0.5	-0.06	5.19	5.19	5.18	95.2	95.5	20.62	4.07	3.97	3.96	0.1	0.1	47.75	2.60	2.58	2.59	0.0	0.0
2.0	-0.06	5.19	5.19	5.18	95.2	95.5	79.48	3.62	3.65	3.64	0.0	0.0	171.66	6.33	4.32	5.98	0.0	0.0
Partially Conditional Model, $\sigma^2 = 0.5$																		
0.0	-0.06	5.19	5.19	5.18	95.2	95.5	2.65	4.20	4.17	4.17	89.2	88.9	17.53	2.75	2.68	2.67	0.0	0.0
0.5	-0.06	5.19	5.19	5.18	95.2	95.5	20.99	3.93	3.91	3.90	0.0	0.0	37.86	2.37	2.35	2.33	0.0	0.0
2.0	-0.06	5.19	5.19	5.18	95.2	95.5	74.06	3.64	3.58	3.60	0.0	0.0	85.02	4.08	2.60	3.93	0.0	0.0

ASE^1 and ASE^2 are the average of naive standard error and robust standard error, respectively; ECP^1 and ECP^2 are the empirical coverage probabilities of nominal 95% confidence interval (×100) based on naive and robust standard errors, respectively.

Table S2.2 Empirical frequency of estimate for treatment effect when omitting covariate Z in the assumed marginal or partially conditional rate functions for the recurrent event following a Markov process; $Z|X \sim N(1+\theta_1 X, \sigma^2)$; $n = 1000$ and $nsim = 1000$; $\alpha = \log 1.05$; all numbers for BIAS, ESE, ASE and ECP (×100) in the table.

	$\zeta = 0$						$\zeta = \log 1.5$						$\zeta = \log 3.0$					
θ_1	BIAS	ESE	ASE[1]	ASE[2]	ECP[1]	ECP[2]	BIAS	ESE	ASE[1]	ASE[2]	ECP[1]	ECP[2]	BIAS	ESE	ASE[1]	ASE[2]	ECP[1]	ECP[2]
Marginal Model, $\sigma^2 = 0.1$																		
0.0	-1.37	5.38	5.13	5.34	92.7	94.0	-1.63	4.53	4.12	4.48	91.6	93.9	-2.89	3.97	2.74	3.97	71.5	88.1
0.5	-1.37	5.38	5.13	5.34	92.7	94.0	19.84	4.33	3.89	4.27	0.4	0.4	57.19	3.59	2.36	3.61	0.0	0.0
2.0	-1.37	5.38	5.13	5.34	92.7	94.0	85.94	3.79	3.36	3.77	0.0	0.0	213.14	3.62	2.32	3.56	0.0	0.0
Marginal Model, $\sigma^2 = 0.5$																		
0.0	-1.37	5.38	5.13	5.34	92.7	94.0	-1.99	4.76	4.04	4.80	88.6	93.4	1.77	5.59	2.49	5.70	59.0	95.2
0.5	-1.37	5.38	5.13	5.34	92.7	94.0	19.54	4.58	3.81	4.60	0.3	0.9	52.24	5.20	2.22	5.19	0.0	0.0
2.0	-1.37	5.38	5.13	5.34	92.7	94.0	85.88	4.19	3.29	4.15	0.0	0.0	172.66	4.61	2.02	4.63	0.0	0.0
Partially Conditional Model, $\sigma^2 = 0.1$																		
0.0	-0.19	5.21	5.19	5.17	95.1	94.9	0.83	4.19	4.19	4.18	93.5	93.4	6.10	2.94	2.80	2.94	40.9	44.5
0.5	-0.19	5.21	5.19	5.17	95.1	94.9	20.64	3.95	3.91	3.90	0.1	0.1	48.80	2.60	2.40	2.64	0.0	0.0
2.0	-0.19	5.21	5.19	5.17	95.1	94.9	79.62	3.63	3.56	3.55	0.0	0.0	168.14	4.68	3.57	4.57	0.0	0.0
Partially Conditional Model, $\sigma^2 = 0.5$																		
0.0	-0.19	5.21	5.19	5.17	95.1	94.9	2.83	4.08	4.11	4.11	88.6	88.7	16.56	3.02	2.52	3.03	0.0	0.0
0.5	-0.19	5.21	5.19	5.17	95.1	94.9	21.09	3.83	3.83	3.83	0.1	0.1	39.22	2.74	2.24	2.75	0.0	0.0
2.0	-0.19	5.21	5.19	5.17	95.1	94.9	74.20	3.55	3.47	3.51	0.0	0.0	97.80	3.50	2.49	3.47	0.0	0.0

ASE[1] and ASE[2] are the average of naive standard error and robust standard error, respectively; ECP[1] and ECP[2] are the empirical coverage probabilities of nominal 95% confidence interval (×100) based on naive and robust standard errors, respectively.

Online Resource 3: Derivation of $\mathcal{B}$ matrix under a misspecified marginal and partially conditional model

3.1 Derivation of $\mathcal{B}$ matrix under a misspecified marginal model

In Appendix 1, we've shown that $\mathcal{B}(\beta) = E\left(w_i(\beta)w_i'(\beta)\right)$, and

$$w_i(\beta) = \int_0^\infty Y_i(t)\left(X_i - \frac{s^{(1)}(\beta,t)}{s^{(0)}(\beta,t)}\right)\left(dN_i(t) - \frac{\exp(\beta X_i)}{s^{(0)}(\beta,t)}d\bar{F}(t)\right) .$$

Therefore

$$\begin{aligned}\mathcal{B}(\beta) = E\Big\{ &\int_0^\infty Y_i(t)\left(X_i - \Delta(\beta)\right)\left(dN_i(t) - \frac{\exp(\beta X_i)}{s^{(0)}(\beta,t)}d\bar{F}(t)\right) \\ &\times \int_0^\infty Y_i(s)\left(X_i - \Delta(\beta)\right)\left(dN_i(s) - \frac{\exp(\beta X_i)}{s^{(0)}(\beta,s)}d\bar{F}(s)\right)\Big\} \\ = \ & E\left\{\int_0^\infty Y_i(t)\left(X_i - \Delta(\beta)\right)^2 \operatorname{Var}\left(dN_i(t)\middle|Y_i(t), X_i, Z_i\right)\right\} \\ &+E\left\{\int_0^\infty\int_0^\infty Y_i(t)Y_i(s)\cdot\left(X_i - \Delta(\beta)\right)^2 \cdot E\left[dN_i(t)dN_i(s)\middle|Y_i(t), Y_i(s), X_i, Z_i\right]\right\} \\ &-E\left\{\int_0^\infty\int_0^\infty Y_i(t)Y_i(s)\cdot\left(X_i - \Delta(\beta)\right)^2 \cdot E\left[dN_i(t)\frac{\exp(\beta X_i)}{s^{(0)}(\beta,s)}d\bar{F}(s)\middle|Y_i(t), Y_i(s), X_i, Z_i\right]\right\} \\ &-E\left\{\int_0^\infty\int_0^\infty Y_i(t)Y_i(s)\cdot\left(X_i - \Delta(\beta)\right)^2 \cdot E\left[dN_i(s)\frac{\exp(\beta X_i)}{s^{(0)}(\beta,t)}d\bar{F}(t)\middle|Y_i(t), Y_i(s), X_i, Z_i\right]\right\} \\ &+E\left\{\int_0^\infty\int_0^\infty Y_i(t)Y_i(s)\cdot\left(X_i - \Delta(\beta)\right)^2 \cdot \frac{\exp(2\beta X_i)}{s^{(0)}(\beta,t)s^{(0)}(\beta,s)}d\bar{F}(t)d\bar{F}(s)\right\} \qquad \text{(S3.1)}\end{aligned}$$

For the first term in (S3.1),

$$\begin{aligned}&E\left\{\int_0^\infty Y_i(t)\left(X_i - \Delta(\beta)\right)^2 \operatorname{Var}\left(dN_i(t)|Y_i(t), X_i, Z_i\right)\right\} \\ &= E\left\{\int_0^\infty Y_i(t)\left(X_i - \Delta(\beta)\right)^2 e^{\eta X_i + \zeta Z_i}d\mu_0(t)\right\} \\ &= \int_0^A \mathcal{G}(t)E\left\{\left(X_i - \Delta(\beta)\right)^2 e^{\eta X_i + \zeta Z_i}\right\}d\mu_0(t) . \qquad \text{(S3.2)}\end{aligned}$$

For the second term in (S3.1), based on the independent increments property of Poisson processes,

$$\begin{aligned}&E\left\{\int_0^\infty\int_0^\infty Y_i(t)Y_i(s)\cdot\left(X_i - \Delta(\beta)\right)^2 \cdot E\left[dN_i(t)dN_i(s)|Y_i(t), Y_i(s), X_i, Z_i\right]\right\} \\ &= E\left\{\int_0^\infty\int_0^\infty Y_i(t)Y_i(s)\cdot\left(X_i - \Delta(\beta)\right)^2 \cdot e^{2(\eta X_i + \zeta Z_i)}d\mu_0(t)d\mu_0(s)\right\} \\ &= \int_0^\infty\int_0^\infty E\left[Y_i(t)Y_i(s)\right]\cdot E\left\{\left(X_i - \Delta(\beta)\right)^2 \cdot e^{2(\eta X_i + \zeta Z_i)}\right\}d\mu_0(t)d\mu_0(s) . \qquad \text{(S3.3)}\end{aligned}$$

The third term and the fourth term are actually the same, so

$$
\begin{aligned}
&E\left\{\int_0^\infty\int_0^\infty Y_i(t)Y_i(s)\cdot(X_i-\mathit{\Delta}(\beta))^2\cdot E\left[dN_i(t)\frac{\exp(\beta X_i)}{s^{(0)}(\beta,s)}d\bar{F}(s)|Y_i(t),Y_i(s),X_i,Z_i\right]\right\}\\
&=E\left\{\int_0^\infty\int_0^\infty Y_i(t)Y_i(s)\cdot(X_i-\mathit{\Delta}(\beta))^2\cdot\frac{\exp(\beta X_i)}{s^{(0)}(\beta,s)}\,d\bar{F}(s)\,e^{\eta X_i+\zeta Z_i}d\mu_0(t)\right\}\\
&=\int_0^\infty\int_0^\infty E\left[Y_i(t)Y_i(s)\right]\cdot E\left\{(X_i-\mathit{\Delta}(\beta))^2\cdot e^{\beta X_i+\eta X_i+\zeta Z_i}\right\}\cdot\frac{E[e^{\eta X_i+\zeta Z_i}]}{E[e^{\beta X_i}]}\,d\mu_0(t)d\mu_0(s)\ , \quad \text{(S3.4)}
\end{aligned}
$$

and the last term is

$$
\begin{aligned}
&E\left\{\int_0^\infty\int_0^\infty Y_i(t)Y_i(s)\cdot(X_i-\mathit{\Delta}(\beta))^2\cdot\frac{\exp(2\beta X_i)}{s^{(0)}(\beta,t)s^{(0)}(\beta,s)}d\bar{F}(t)d\bar{F}(s)\right\}\\
&=E\left\{\int_0^\infty\int_0^\infty Y_i(t)Y_i(s)\cdot(X_i-\mathit{\Delta}(\beta))^2\cdot\frac{\exp(2\beta X_i)}{\left[E(e^{\beta X_i})\right]^2}\left[E(e^{\eta X_i+\zeta Z_i})\right]^2d\mu_0(t)d\mu_0(s)\right\}\\
&=\int_0^\infty\int_0^\infty E\left[Y_i(t)Y_i(s)\right]\cdot E\left[(X_i-\mathit{\Delta}(\beta))^2\cdot e^{2\beta X_i}\right]\left(\frac{E[e^{\eta X_i+\zeta Z_i}]}{E[e^{\beta X_i}]}\right)^2 d\mu_0(t)d\mu_0(s)\ . \quad \text{(S3.5)}
\end{aligned}
$$

Equations (S3.3) to (S3.5) are all of the form,

$$
\int_0^\infty\int_0^\infty E[Y_i(t)Y_i(s)]\cdot H(\beta,\eta,\zeta)\,d\mu_0(t)d\mu_0(s)\ ,
$$

where $H(\beta,\eta,\zeta)$ is the corresponding deterministic function of the parameters (β,η,ζ). For example, in (S3.3),

$$
H(\beta,\eta,\zeta)=E\left\{(X_i-\mathit{\Delta}(\beta))^2\cdot e^{2(\eta X_i+\zeta Z_i)}\right\}\ .
$$

In order to evaluate (S3.3) to (S3.5), we need to get the expression for

$$
\int_0^\infty\int_0^\infty E[Y_i(t)Y_i(s)]\,d\mu_0(t)d\mu_0(s)\ .
$$

Note that $E[Y_i(t)Y_i(s)]\neq P(C_i>s)P(C_i>t)$ because at risk indicator for an individual at different times are correlated, so

$$\int_0^\infty\int_0^\infty E[Y_i(t)Y_i(s)]\,d\mu_0(t)d\mu_0(s) = \int_0^A\int_0^A P(C_i > t, C_i > s)\,d\mu_0(t)d\mu_0(s)$$

$$= \int_0^A\int_0^s P(C_i > s)\,d\mu_0(t)d\mu_0(s) + \int_0^A\int_s^A P(C_i > t)\,d\mu_0(t)d\mu_0(s)$$

$$= \int_0^A \mathcal{G}(s)\mu_0(s)d\mu_0(s) + \int_0^A\int_0^t \mathcal{G}(t)d\mu_0(s)d\mu_0(t)$$

$$= 2\cdot\int_0^A \mathcal{G}(s)\mu_0(s)d\mu_0(s) \overset{def}{=} Q\,. \tag{S3.6}$$

Substituting (S3.6) in (S3.3) to (S3.5), we obtain

$$\mathcal{B}(\beta) = \left(\int_0^\infty \mathcal{G}(t)d\mu_0(t)\right)\cdot E\left[(X_i - \Delta(\beta))^2 e^{\eta X_i+\zeta Z_i}\right] + \ Q\times\Bigg[E\left\{(X_i-\Delta(\beta))^2\cdot e^{2(\eta X_i+\zeta Z_i)}\right\}$$
$$-2\times E\left\{(X_i-\Delta(\beta))^2\cdot e^{\beta X_i+\eta X_i+\zeta Z_i}\right\}\cdot\frac{E[e^{\eta X_i+\zeta Z_i}]}{E[e^{\beta X_i}]}$$
$$+\,E\left\{(X_i-\Delta(\beta))^2\cdot e^{2\beta X_i}\right\}\left(\frac{E[e^{\eta X_i+\zeta Z_i}]}{E[e^{\beta X_i}]}\right)^2\Bigg]\,. \tag{S3.7}$$

3.2 Derivation of $\tilde{\mathcal{B}}$ matrix under a misspecified partially conditional model

In Appendix 2, we knew that

$$\tilde{\mathcal{B}}(\beta) = E[\tilde{w}_i(\beta)\tilde{w}_i'(\beta)]\,,$$

where

$$\tilde{w}_i(\beta) = \sum_{k=1}^\infty \tilde{w}_{ik}(\beta) = \sum_{k=1}^\infty\int_0^\infty \bar{Y}_{ik}(s)\left(X_i - \frac{s_k^{(1)}(\beta,s)}{s_k^{(0)}(\beta,s)}\right)\cdot\left(dN_{ik}(s) - \frac{e^{\beta X_i}}{s_k^{(0)}(\beta,s)}\,d\bar{F}_k(s)\right)\,,$$

and $d\bar{F}_k(s) = E[\bar{Y}_{ik}(s)dN_{ik}(s)] = E[\bar{Y}_{ik}(s)d\mu_i(s)] = s_k^{(0)}(s)ds$. Therefore,

$$\tilde{\mathcal{B}}(\beta) = E\left[\sum_{j,k}\tilde{w}_{ij}(\beta)\tilde{w}_{ik}(\beta)\right] = \sum_{j=1}^\infty E\left[\tilde{w}_{ij}^2(\beta)\right] + \sum_{j\neq k} E\left[\tilde{w}_{ij}(\beta)\tilde{w}_{ik}(\beta)\right] \overset{def}{=} B_1 + B_2\,.$$

Let $\Delta_j(\beta,t) = s_j^{(1)}(\beta,t)/s_j^{(0)}(\beta,t)$, then B_1 can be written as

$$
\begin{aligned}
B_1 &= \sum_{j=1}^{\infty} E\left[w_{ij}^2(\beta)\right] = \sum_{j=1}^{\infty} E\left[\int_0^{\infty} \bar{Y}_{ij}(t)\left(X_i - \Delta_j(\beta,t)\right)^2 \left(dN_{ij}(t)\right)^2\right] \\
&= \sum_{j=1}^{\infty} E\left[\int_0^{\infty} \bar{Y}_{ij}(t)\left(X_i - \Delta_j(\beta,t)\right)^2 \operatorname{Var}\left(dN_{ij}(t)|\bar{Y}_{ij}(t), X_i, Z_i\right)\right] \\
&= \sum_{j=1}^{\infty} E\left[\int_0^{\infty} \bar{Y}_{ij}(t)\left(X_i - \Delta_j(\beta,t)\right)^2 d\mu_i(t)\right] \\
&= \sum_{j=1}^{\infty} E\left[\int_0^{A} \mathcal{G}(t)\cdot P(Y_{ij}(t)=1)\cdot\left(X_i - \Delta_j(\beta,t)\right)^2 d\mu_i(t)\right] . \qquad \text{(S3.8)}
\end{aligned}
$$

For the second part of $\tilde{\mathcal{B}}(\beta)$,

$$
\begin{aligned}
B_2 &= \sum_{j\neq k} E[\tilde{w}_{ij}(\beta)\tilde{w}_{ik}(\beta)] = 2 * \sum_{j>k} E[w_{ij}(\beta)w_{ik}(\beta)] \\
&= 2 * \Bigg\{ E\left[\sum_{j>k}\int_0^{\infty}\int_0^{\infty} \bar{Y}_{ij}(t)\bar{Y}_{ik}(s)\left(X_i - \Delta_j(\beta,t)\right)\left(X_i - \Delta_k(\beta,s)\right) dN_{ij}(t)dN_{ik}(s)\right] \\
&\quad - E\left[\sum_{j>k}\int_0^{\infty}\int_0^{\infty} \bar{Y}_{ij}(t)\bar{Y}_{ik}(s)\left(X_i - \Delta_j(\beta,t)\right)\left(X_i - \Delta_k(\beta,s)\right) \frac{e^{\beta X_i}}{s_k^{(0)}(\beta,s)} dN_{ij}(t)d\bar{F}_k(s)\right] \\
&\quad - E\left[\sum_{j>k}\int_0^{\infty}\int_0^{\infty} \bar{Y}_{ij}(t)\bar{Y}_{ik}(s)\left(X_i - \Delta_j(\beta,t)\right)\left(X_i - \Delta_k(\beta,s)\right) \frac{e^{\beta X_i}}{s_j^{(0)}(\beta,t)} dN_{ik}(s)d\bar{F}_j(t)\right] \\
&\quad + E\left[\sum_{j>k}\int_0^{\infty}\int_0^{\infty} \bar{Y}_{ij}(t)\bar{Y}_{ik}(s)\left(X_i - \Delta_j(\beta,t)\right)\left(X_i - \Delta_k(\beta,s)\right) \frac{e^{2\beta X_i}}{s_j^{(0)}(\beta,t)\cdot s_k^{(0)}(\beta,s)} d\bar{F}_j(t)d\bar{F}_k(s)\right] \Bigg\} .
\end{aligned}
$$

In fact, $j > k$ implies $t > s$. Recall that $d\bar{F}_k(s) = s_k^{(0)}(s)ds$, we can write

$$
\begin{aligned}
B_2 &= 2 * \Bigg\{ \sum_{j>k} E\left[\int_0^{\infty}\int_0^{t} \bar{Y}_{ij}(t)\bar{Y}_{ik}(s)\left(X_i - \Delta_j(\beta,t)\right)\left(X_i - \Delta_k(\beta,s)\right) dN_{ik}(s)dN_{ij}(t)\right] \\
&\quad - \sum_{j>k} E\left[\int_0^{\infty}\int_0^{t} \bar{Y}_{ij}(t)\bar{Y}_{ik}(s)\left(X_i - \Delta_j(\beta,t)\right)\left(X_i - \Delta_k(\beta,s)\right) \frac{e^{\beta X_i} s_k^{(0)}(s)}{s_k^{(0)}(\beta,s)} ds dN_{ij}(t)\right] \\
&\quad - \sum_{j>k} E\left[\int_0^{\infty}\int_0^{t} \bar{Y}_{ij}(t)\bar{Y}_{ik}(s)\left(X_i - \Delta_j(\beta,t)\right)\left(X_i - \Delta_k(\beta,s)\right) \frac{e^{\beta X_i} s_j^{(0)}(t)}{s_j^{(0)}(\beta,t)} dN_{ik}(s)dt\right] \\
&\quad + \sum_{j>k} E\left[\int_0^{\infty}\int_0^{t} \bar{Y}_{ij}(t)\bar{Y}_{ik}(s)\left(X_i - \Delta_j(\beta,t)\right)\left(X_i - \Delta_k(\beta,s)\right) \frac{e^{2\beta X_i} s_j^{(0)}(t)\, s_k^{(0)}(s)}{s_j^{(0)}(\beta,t)\cdot s_k^{(0)}(\beta,s)} ds dt\right] \Bigg\} \\
&\overset{\text{def}}{=} 2 * \sum_{j>k}(b_{21} - b_{22} - b_{23} + b_{24}) .
\end{aligned}
$$

The (conditional) expectations with respect to $\{X_i, Z_i, Y_i(t), Y_{ik}(s), Y_{ij}(t), dN_{ik}(s), dN_{ij}(t)\}$ must be taken in a way that respects the time ordering. Note that

$$\begin{aligned} b_{21} &= E\left[\int_0^\infty\int_0^t \bar{Y}_{ij}(t)\bar{Y}_{ik}(s)\left(X_i-\Delta_j(\beta,t)\right)\left(X_i-\Delta_k(\beta,s)\right)dN_{ik}(s)dN_{ij}(t)\right] \\ &= E\left[\int_0^\infty\int_0^t \bar{Y}_{ij}(t)\bar{Y}_{ik}(s)\left(X_i-\Delta_j(\beta,t)\right)\left(X_i-\Delta_k(\beta,s)\right)dN_{ik}(s)d\mu_i(t)\right] \\ &= E\left[\int_0^\infty\int_0^t Y_i(t)\bar{Y}_{ik}(s)\left(X_i-\Delta_j(\beta,t)\right)\left(X_i-\Delta_k(\beta,s)\right)dN_{ik}(s)P(Y_{ij}(t)=1|dN_{ik}(s),\bar{Y}_{ik}(s))d\mu_i(t)\right] \\ &= E\left[\int_0^\infty\int_0^t Y_i(t)\bar{Y}_{ik}(s)\left(X_i-\Delta_j(\beta,t)\right)\left(X_i-\Delta_k(\beta,s)\right)P(Y_{ij}(t)=1|dN_{ik}(s)=1,Y_{ik}(s))d\mu_i(s)d\mu_i(t)\right] \\ &= E\Big[\int_0^A\int_0^t \mathcal{G}(t)\left(X_i-\Delta_j(\beta,t)\right)\left(X_i-\Delta_k(\beta,s)\right)P(Y_{ik}(s)=1)P(Y_{ij}(t)=1|dN_{ik}(s)=1,Y_{ik}(s)=1)d\mu_i(s)d\mu_i(t)\Big]\,. \end{aligned}$$

$$\begin{aligned} b_{22} &= E\left[\int_0^\infty\int_0^t \bar{Y}_{ij}(t)\bar{Y}_{ik}(s)\left(X_i-\Delta_j(\beta,t)\right)\left(X_i-\Delta_k(\beta,s)\right)\frac{e^{\beta X_i}s_k^{(0)}(s)}{s_k^{(0)}(\beta,s)}dsdN_{ij}(t)\right] \\ &= E\left[\int_0^\infty\int_0^t \bar{Y}_{ij}(t)\bar{Y}_{ik}(s)\left(X_i-\Delta_j(\beta,t)\right)\left(X_i-\Delta_k(\beta,s)\right)\frac{e^{\beta X_i}s_k^{(0)}(s)}{s_k^{(0)}(\beta,s)}dsd\mu_i(t)\right] \\ &= E\left[\int_0^A\int_0^t \mathcal{G}(t)\left(X_i-\Delta_j(\beta,t)\right)\left(X_i-\Delta_k(\beta,s)\right)\frac{e^{\beta X_i}s_k^{(0)}(s)}{s_k^{(0)}(\beta,s)}P(Y_{ik}(s)=1)P(Y_{ij}(t)=1|Y_{ik}(s)=1)dsd\mu_i(t)\right] \end{aligned}$$

$$\begin{aligned} b_{23} &= E\left[\int_0^\infty\int_0^t \bar{Y}_{ij}(t)\bar{Y}_{ik}(s)\left(X_i-\Delta_j(\beta,t)\right)\left(X_i-\Delta_k(\beta,s)\right)\frac{e^{\beta X_i}s_j^{(0)}(t)}{s_j^{(0)}(\beta,t)}dN_{ik}(s)dt\right] \\ &= E\left[\int_0^\infty\int_0^t Y_i(t)\bar{Y}_{ik}(s)\left(X_i-\Delta_j(\beta,t)\right)\left(X_i-\Delta_k(\beta,s)\right)\frac{e^{\beta X_i}s_j^{(0)}(t)}{s_j^{(0)}(\beta,t)}P(Y_{ij}(t)=1|dN_{ik}(s),Y_{ik}(s))dN_{ik}(s)dt\right] \\ &= E\left[\int_0^\infty\int_0^t Y_i(t)\bar{Y}_{ik}(s)\left(X_i-\Delta_j(\beta,t)\right)\left(X_i-\Delta_k(\beta,s)\right)\frac{e^{\beta X_i}s_j^{(0)}(t)}{s_j^{(0)}(\beta,t)}P(Y_{ij}(t)=1|dN_{ik}(s)=1,Y_{ik}(s))d\mu_i(s)dt\right] \\ &= E\left[\int_0^\infty\int_0^t Y_i(t)\left(X_i-\Delta_j(\beta,t)\right)\left(X_i-\Delta_k(\beta,s)\right)\frac{e^{\beta X_i}s_j^{(0)}(t)}{s_j^{(0)}(\beta,t)}P(Y_{ij}(t)=1|dN_{ik}(s)=1,Y_{ik}(s)=1)P(Y_{ik}(s)=1)d\mu_i(s)dt\right] \\ &= E\left[\int_0^A\int_0^t \mathcal{G}(t)\left(X_i-\Delta_j(\beta,t)\right)\left(X_i-\Delta_k(\beta,s)\right)\frac{e^{\beta X_i}s_j^{(0)}(t)}{s_j^{(0)}(\beta,t)}P(Y_{ij}(t)=1|dN_{ik}(s)=1,Y_{ik}(s)=1)P(Y_{ik}(s)=1)d\mu_i(s)dt\right] \end{aligned}$$

$$b_{24} = E\left[\int_0^{\infty}\int_0^{t} \bar{Y}_{ij}(t)\bar{Y}_{ik}(s)\left(X_i - \varDelta_j(\beta,t)\right)\left(X_i - \varDelta_k(\beta,s)\right)\frac{e^{2\beta X_i} s_j^{(0)}(t) s_k^{(0)}(s)}{s_j^{(0)}(\beta,t)\cdot s_k^{(0)}(\beta,s)}\,dsdt\right]$$
$$= E\left[\int_0^{A}\int_0^{t} \left(X_i - \varDelta_j(\beta,t)\right)\left(X_i - \varDelta_k(\beta,s)\right)\frac{e^{2\beta X_i} s_j^{(0)}(t) s_k^{(0)}(s)}{s_j^{(0)}(\beta,t)\cdot s_k^{(0)}(\beta,s)}\mathcal{G}(t)P(Y_{ij}(t)=1, Y_{ik}(s)=1)\,dsdt\right]$$
$$= E\left[\int_0^{A}\int_0^{t} \mathcal{G}(t)\left(X_i - \varDelta_j(\beta,t)\right)\left(X_i - \varDelta_k(\beta,s)\right)\frac{e^{2\beta X_i} s_j^{(0)}(t) s_k^{(0)}(s)}{s_j^{(0)}(\beta,t)\cdot s_k^{(0)}(\beta,s)}P(Y_{ik}(s)=1)P(Y_{ij}(t)=1|Y_{ik}(s)=1)\,dsdt\right]$$

Therefore, we have

$$B_2 = 2*\sum_{j>k} E\Bigg\{\int_0^{A}\int_0^{t} \mathcal{G}(t)\cdot\left(X_i - \varDelta_j(\beta,t)\right)\cdot\left(X_i - \varDelta_k(\beta,s)\right)\cdot P(Y_{ik}(s)=1) \tag{S3.9}$$
$$\times\Big[P(Y_{ij}(t)=1|dN_{ik}(s)=1, Y_{ik}(s)=1)\rho_i(s)\rho_i(t) - \frac{e^{\beta X_i} s_k^{(0)}(s)}{s_k^{(0)}(\beta,s)}P(Y_{ij}(t)=1|Y_{ik}(s)=1)\rho_i(t)$$
$$-\frac{e^{\beta X_i} s_j^{(0)}(t)}{s_j^{(0)}(\beta,t)}P(Y_{ij}(t)=1|dN_{ik}(s)=1, Y_{ik}(s)=1)\rho_i(s) + \frac{e^{2\beta X_i} s_j^{(0)}(t) s_k^{(0)}(s)}{s_j^{(0)}(\beta,t)\cdot s_k^{(0)}(\beta,s)}P(Y_{ij}(t)=1|Y_{ik}(s)=1)\Big]dsdt\Bigg\}$$

which alternatively can be written as

$$B_2 = 2*\sum_{j>k} E\Bigg\{\int_0^{A}\int_0^{t} \mathcal{G}(t)\cdot\left(X_i - \varDelta_j(\beta,t)\right)\cdot\left(X_i - \varDelta_k(\beta,s)\right)\cdot P(Y_{ik}(s)=1)\cdot\left(\rho_i(t) - \frac{e^{\beta X_i} s_j^{(0)}(t)}{s_j^{(0)}(\beta,t)}\right)$$
$$\times\left(P(Y_{ij}(t)=1|dN_{ik}(s)=1, Y_{ik}(s)=1)\rho_i(s) - \frac{e^{\beta X_i} s_k^{(0)}(s)}{s_k^{(0)}(\beta,s)}P(Y_{ij}(t)=1|Y_{ik}(s)=1)\right)dsdt\Bigg\}. \tag{S3.10}$$

Calculation of $\tilde{\mathcal{B}}(\beta)$ requires probabilities $P(Y_{ik}(s)=1)$, $P(Y_{ij}(t)|dN_{ik}(s)=1, Y_{ik}(s)=1)$ and $P(Y_{ij}(t)=|Y_{ik}(s)=1)$. Under the Poisson model we have

$$P(Y_{ik}(s)=1) = P(N_i(s^-)=k-1) = \frac{e^{-\mu_i(s)}(\mu_i(s))^{k-1}}{(k-1)!} \tag{S3.11}$$

$$\begin{aligned} P(Y_{ij}(t)=1|Y_{ik}(s)=1) &= P(N_i(t^-)=j-1|N_i(s^-)=k-1) \\ &= P(N_i(s,t^-)=j-k) \\ &= \frac{e^{-[\mu_i(t)-\mu_i(s)]}(\mu_i(t)-\mu_i(s))^{j-k}}{(j-k)!} \end{aligned} \tag{S3.12}$$

$$\begin{aligned} P(Y_{ij}(t)=1|dN_{ik}(s)=1, Y_{ik}(s)=1) &= P(N_i(t^-)=j-1|dN_{ik}(s)=1, N_i(s^-)=k-1) \\ &= P(N_i(t^-)=j-1|N_i(s)=k) \\ &= P(N_i(s^+,t^-)=j-k-1) \\ &= \frac{e^{-[\mu_i(t)-\mu_i(s)]}(\mu_i(t)-\mu_i(s))^{j-k-1}}{(j-k-1)!} \end{aligned} \tag{S3.13}$$
